# Modulus Matching and Interface Enhancement: A Synergistic Strategy for Antidelamination High‐Performance Stretchable Triboelectric Nanogenerator

**DOI:** 10.1002/advs.202518027

**Published:** 2025-11-19

**Authors:** Shiwei Xu, Pengfan Wu, Feng Qin, Endian Cui, Zhongyong Mo, Hengyu Guo, Xiaojing Mu, Hua Yu

**Affiliations:** ^1^ Key Laboratory of Optoelectronic Technology & Systems Ministry of Education International R&D Center of Micro‐Nano Systems and New Materials Technology Chongqing University Chongqing 400044 China; ^2^ State Key Laboratory of Mechanical Transmission College of Mechanical and Vehicle Engineering Chongqing University Chongqing 400044 China

**Keywords:** antidelamination, homologous polymer pairing, interfacial toughness, stretchable electronics, triboelectric nanogenerator

## Abstract

With the growing demand for flexible self‐powered energy sources for wearable bioelectronics, triboelectric nanogenerator (TENG) have emerged as a promising technology for harvesting biomechanical energy. However, interfacial delamination between functional layers caused by mechanical mismatch has limited their practical application. Existing strategies struggle to balance bond strength, electrical conductivity, and stretchability. Inspired by molecularly continuous interfaces in biological systems, this study proposes a novel TENG construction strategy based on homologous polymer pairing. The triboelectric layer is a tailored thermoplastic polyurethane (TPU)/polyvinyl chloride (PVC)/ dibutyl adipate (DBA) gel (TPD‐gel) with high electronegativity and tunable plasticity, while the electrode consists of a carbon nanotube‐embedded TPU microfiber network that maintains stable conductivity under 200% strain. Both layers share a polyurethane matrix, enabling modulus matching (ratio < 2) and strain coordination, which fundamentally suppresses stress concentration. Through ultrasonic cavitation treatment, the interfacial toughness is significantly enhanced to 190 N m^−1^, which is 3.2 times that of conventional PDMS‐based interfaces. The resulting TENG exhibits high triboelectric charge density, strain‐insensitive conductivity, and over 100% extensibility. Furthermore, a delamination‐resistant triboelectric sensor integrated with a deep learning algorithm achieves 97.8% accuracy in character recognition, demonstrating potential for wearable health monitoring and human–machine interaction.

## Graphical abstract

A bioinspired TENG features a homologously paired interface with matched modulus. Ultrasonic cavitation enhances its toughness to 190 N m^−1^ (3.2× higher), preventing delamination under 400% strain.

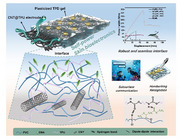

## 1 Introduction

The rapid development of ergonomic bioelectronic devices, such as continuous health monitoring systems, smart prosthetics, and augmented reality interfaces, has fueled a growing demand for autonomous energy sources capable of operating seamlessly within human biomechanical environments.^[^ ^1^, ^2^, ^3^, ^4^ ^]^ Traditional batteries, limited by their short service life, rigid structure, and frequent need for recharging, are fundamentally incompatible with the dynamic nature of wearable systems.^[^ ^5^, ^6^, ^7^, ^8^ ^]^ Triboelectric nanogenerator (TENG), which harvests energy from human motion through contact electrification and electrostatic induction, offers a compelling solution.^[^ ^9^, ^10^, ^11^ ^]^ Their intrinsic flexibility, lightweight construction, and material diversity make TENG ideal power sources for the next generation of epidermal sensors, tactile interfaces, and biomedical devices.^[^ ^12^, ^13^, ^14^, ^15^, ^16^ ^]^

However, a critical challenge hindering the practical application of TENG is interfacial delamination caused by mechanical mismatch. This failure mechanism originates from the disparate mechanical properties of different functional layers.^[^ ^17^, ^18^, ^19^ ^]^ Conventional TENG architectures typically combine rigid conductive electrodes, such as metal thin films or carbon nanotubes, with soft triboelectric elastomers like polydimethylsiloxane or Ecoflex. During dynamic deformation, including stretching over 100% strain, twisting, or repeated bending, the significant modulus mismatch, for instance 1–10 GPa versus 0.1–10 MPa, generates substantial shear stress at the interface. This stress concentration initiates progressive delamination, where cracks propagate along weak interfacial regions such as physisorbed areas under cyclic loading, with interfacial toughness *Γ* below 10 J m^−^^2^, ultimately resulting in complete detachment and total device failure. Moreover, even intrinsically stretchable conductors like hydrogels or liquid metal networks face difficulty in forming strong adhesion with triboelectric layers. For example, the interfacial adhesion energy between hydrogels and polydimethylsiloxane, PDMS, is typically less than 5 J m^−^^2^, well below the 50 to 500 J m^−^^2^ required for stable integration under physiological motion conditions.^[^ ^20^, ^21^ ^]^ These limitations fundamentally restrict the long‐term reliability and energy conversion efficiency of wearable triboelectric generators.

To address these issues, current strategies focus on the following aspects. Physical interlocking, which relies on micropillar arrays or interpenetrating nanofibers, can enhance mechanical fixation but is limited in scalability by complex fabrication processes.^[^ ^22^ ^]^ Chemical bonding strategies using covalent linkers such as silane coupling agents improve adhesion, yet often compromise the reconfigurability of the materials.^[^ ^23^, ^24^, ^25^ ^]^ The gel bonding strategy employs an adhesive gel to achieve natural and intimate contact between the electrode layer and the triboelectric layer, thereby avoiding the need for additional complex chemical modifications.^[^ ^26^ ^]^ However, this approach is not universally applicable to all material pairs, as it does not guarantee robust adhesion between the gel layer and every type of triboelectric elastomer.^[^ ^27^, ^28^ ^]^ Despite these advances, a comprehensive solution that simultaneously achieves high interfacial toughness (greater than 100 J m^−2^), strain‐insensitive conductivity, stretchability exceeding 100%, and uncompromised triboelectric charge density remains elusive.

Inspired by biological systems possessing molecularly unified interfaces, such as the tendon‐to‐bone junction, we propose a bioinspired strategy to circumvent delamination through the architectural design of a homologous polymer‐based pair.^[^ ^29^ ^]^ This design integrates a plasticized thermoplastic polyurethane (TPU)/polyvinyl chloride (PVC)/dibutyl adipate (DBA) gel (denoted as TPD) as the triboelectric layer with a carbon nanotube (CNT)‐embedded TPU microfiber network (denoted as C@T) as the electrode. In the TPD gel, the DBA plasticizer swells the chains of TPU and PVC, endowing the composite with tunable plasticity. The chlorine‐rich PVC combined with a high DBA content provides strong electron affinity, enhancing the charge density and triboelectric negativity. For the electrode, ultrasonically driven CNT form a robust percolation network on the TPU fiber surface, maintaining excellent conductivity even under 200% strain. Crucially, CNT “rootlets” penetrate the interface, creating a nanomechanical interlock with the gel layer that significantly increases the crack propagation energy. By constructing both functional layers on an identical polyurethane backbone, we achieved an intrinsically matched system with 3D compatibility, ultimately overcoming the fundamental challenge of delamination in TENG. We demonstrate that the interfacial stability in this stretchable TENG is governed by the synergy of modulus matching, strain compatibility, and molecular‐level adhesion. Specifically, this homologous system exhibits an exceptionally low modulus mismatch, with a ratio of less than 2 between the C@T electrode and the 40% TPU–PVC gel, effectively suppressing strain energy concentration at the interface. Moreover, the closely matched fracture elongation between the TPD gel and the C@T electrode ensures superior strain coordination, preventing stress concentration and peeling during deformation. Most importantly, via ultrasonic cavitation treatment, we significantly enhanced the interfacial adhesion, achieving a remarkable toughness of 190 N m^−1^, which is 3.2 times higher than that of conventional PDMS‐based interfaces. Mechanistic studies reveal that this robust adhesion originates from the topological entanglement of polyurethane chains with CNT and ultrasonically enhanced intermolecular interactions. The optimized interface maintains structural integrity without delamination under uniaxial stretching up to 400% and other complex deformations. This work establishes a fundamental principle: homologous polymer systems with chemo‐mechanically synchronized interfaces are essential for developing anti‐delamination, reliable, and high‐performance wearable energy harvesters. The presented C@T‐TPD gel platform not only surmounts a long‐standing obstacle in flexible electronics but also offers a broadly applicable strategy for designing next‐generation wearable devices capable of withstanding extreme physiological motions and harsh environmental conditions.

## 2 Result and Discussion

### 2.1 Design of the C@T‐TPD Gel

Stretchable electronic devices are typically composed of mechanically mismatched components. When subjected to stretching, the significant modulus mismatch between these components induces severe stress concentration at their interfaces, which represents a primary cause of interfacial delamination. From the perspective of fracture mechanics, interfacial delamination is driven by the interfacial energy release rate (*G*).^[^ ^30^ ^]^

(1) $$G=-\frac{\partial \Pi }{\partial A}$$

where *Π* denotes the total potential energy of the system, and *A* represents the area of crack propagation. Physically, *G* quantifies the energy released by the system per unit area of crack advancement. Unstable crack propagation occurs when *G* ≥ *Γ*, where *Γ* denotes the interfacial toughness. In a flexible stretchable triboelectric nanogenerator, the electrode layer and the triboelectric layer can be regarded as two intimately bonded components, which may be simplified using a bilayer beam model. Under this simplification, *G* can be approximated as:

(2) $$G_{I}=\frac{Z\cdot {\left(\varepsilon \right)}^{2}\cdot h}{2}\cdot f\left(E_{1},E_{2},\nu_{1},\nu_{2}\right)$$

where *h* denotes the thickness of the thinner layer, *Z* represents the mismatch parameter defined as $Z=\frac{E_{1}}{1-\nu_{1}^{2}}-\frac{E_{2}}{1-\nu_{2}^{2}}$, and *f(x)* is a material‐dependent correction function.

**Figure 1:**
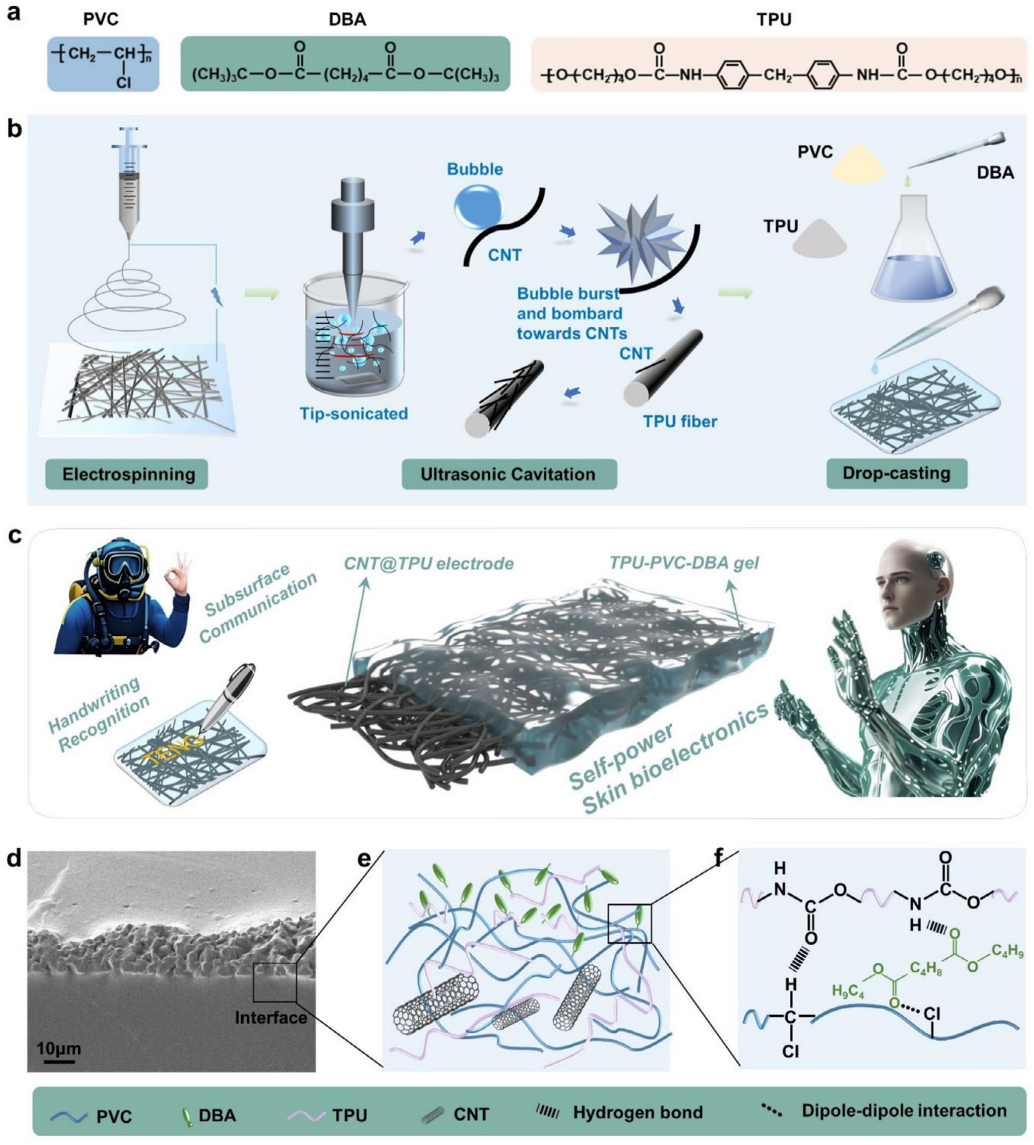
Fabrication of the C@T‐TPD gel. a) The chemical structures of the TPU, PVC, and DBA. b) Schematic preparation procedure of C@T‐TPD gel. c) Schematic structure of the gel‐based self‐powered skin bioelectronics and different application scenarios. d) Cross‐sectional SEM image of C@T‐TPD gel. The scale bars were 10 µm. e) Schematic illustration of the interface between C@T electrode and TPD gel. f) Interaction between various components in TPD gel.

Based on the above analysis, improving the mechanical stability of stretchable triboelectric nanogenerators requires reducing modulus mismatch, enhancing strain compatibility, matching the Poisson's ratio to minimize *G*, and increasing the interfacial toughness to raise *Γ*. We used TPU and PVC plasticized with DBA to form a gel (**Figure** **1**a), which provides the electrode and triboelectric layers with tailored stretchability to reduce modulus mismatch, along with strong interlayer adhesion to improve interfacial toughness. We introduce a simple method for fabricating nanofiber composite gels featuring stretchable conductivity, robust interfacial adhesion, and tunable mechanical properties. This approach is based on a combined manufacturing process involving electrospinning, ultrasonic cavitation treatment, and drop casting, as illustrated in Figure 1b. Briefly, a pre‐prepared TPU solution was electrospun into continuous fibers with random orientation yet dense packing, resulting in a thin, 3D macroporous structure. The TPU mat was then treated in an ultrasonic cavitation bath containing a carbon nanotube suspension, leading to the synthesis of a C@T composite film. During ultrasonication, the collapse of cavitation bubbles generates transient phenomena such as high temperature, microjets, and shock waves. These effects facilitate the impact and attachment of suspended carbon nanotubes onto the electrospun fiber surfaces.^[^ ^31^ ^]^ As shown in Figure S1 (Supporting Information), the SEM and TEM images of C@T electrode clearly show that CNT “rooting” from the TPU fibers and partially embeds or penetrates into the TPU fibers. The Raman spectroscopy shows that the characteristic polymer bands of TPU are almost absent after CNT introduction. Notably, the signature D‐band and 2D‐band of CNT undergo a redshift—from 1593 and 2678 cm^−1^ to 1588 and 2674 cm^−1^, respectively. This spectral shift is indicative of interfacial stress transfer, which can be ascribed to the embedding of carbon nanotubes into the surface of electrospun TPU fibers. As a result, the resulting film exhibits excellent conductivity and outstanding durability even under high strain conditions. Finally, a bilayered C@T‐TPD gel with strain‐adaptive behavior and a strong bonded interface was fabricated by drop casting a TPD solution onto the conductive surface of the C@T film. The resulting gel structure is shown in Figure 1c.

Cross‐sectional SEM images and EDS elemental mapping (Figure 1d and Figure S2, Supporting Information) reveal that during the formation of the plasticized TPD gel, the precursor solution infiltrates and penetrates the 3D C@T layer. The porous electrode structure and the embedded CNT microstructures on the fiber surfaces enhance mechanical interlocking, thereby contributing to improved interfacial toughness. The PVC gel, containing a high amount of DBA plasticizer, exhibits not only a significantly elevated dielectric constant (90 to 300 times higher than that of pure PVC), but also strongly negative triboelectric properties (surpassing even perfluoroalkoxy alkane, PFA), along with remarkable elasticity and durability.^[^ ^32^, ^33^ ^]^ These characteristics make it an ideal triboelectric material for high‐performance stretchable TENG. In this study, a high DBA content with a PVC:DBA ratio of 1:2 was used, which imparts enhanced triboelectric output and a low elastic modulus to the TENG. However, as DBA is a small molecule that lacks chemical bonding with the PVC matrix, it tends to migrate toward the interface over time. This migration significantly reduces the interfacial toughness between the PVC gel and the C@T electrode.^[^ ^34^ ^]^

To address the issue of interfacial mechanical instability caused by migration, we introduced TPU into the PVC gel matrix, forming a ternary TPD gel system. Figure S3 (Supporting Information) shows the FTIR spectra of TPU–PVC composites with different blending ratios. The peak near 3332 cm^−1^ corresponds to the N─H stretching vibration in the urethane group; the peaks at 2962 and 2856 cm^−1^ are attributed to the asymmetric and symmetric stretching vibrations of methylene (─CH_2_), respectively; the peak at 2923 cm^−1^ is assigned to C─H stretching; the region between 1630 and 1860 cm^−1^ shows carbonyl stretching vibrations, with a prominent peak around 1730 cm^−1^; the peak at 1599 cm^−1^ arises from C─C aromatic ring stretching; and the peak near 1532 cm^−1^ is a combined band resulting from N─H bending vibrations of urethane and C─N stretching vibrations. Compared to pure PVC‐DBA, new absorption peaks emerge in the TPD blends at 3332, 1599, and 1532 cm^−1^ with increasing TPU content. The intensity of these characteristic peaks increases with higher TPU loading, while the C─H stretching vibration at 2962 cm^−1^ gradually weakens, indicating weak intermolecular interactions between PVC and TPU. The absorption at 1532 cm^−1^, associated with N─H bending and C─N stretching of the urethane group, broadens and intensifies, and shifts to a higher wavenumber. This change occurs because PVC disrupts the hydrogen bonding within TPU, while new hydrogen bonds form between the carbonyl groups of TPU and the α‐hydrogen of PVC. The observed peak shifts in the infrared spectra demonstrate that the TPD gel undergoes heterochain polymerization via hydrogen bonding between amino and carbonyl groups. The good compatibility between DBA and PVC originates from dipole–dipole interactions between the two carbonyl groups of DBA and the C─Cl bonds of the PVC chains. Additionally, hydrogen bonding occurs through electrostatic attraction between hydrogen in the ─NH─ group of TPU hard segments and the oxygen atoms in the ester groups of DBA, as illustrated in Figure 1f. When blended with PVC, TPU acts similarly to a small‐molecule plasticizer, serving as a polymeric plasticizer for PVC. On one hand, polymeric plasticizers alone exhibit insufficient plasticizing efficiency, which can be enhanced by combination with small‐molecule plasticizers. On the other hand, the mixture of TPU and small‐molecule plasticizers helps attract and immobilize DBA molecules, preventing their migration toward the surface of the PVC gel, as shown in Figure 1e.

### 2.2 Characterization of the Interface between C@T Electrode and TPD Gel

**Figure 2:**
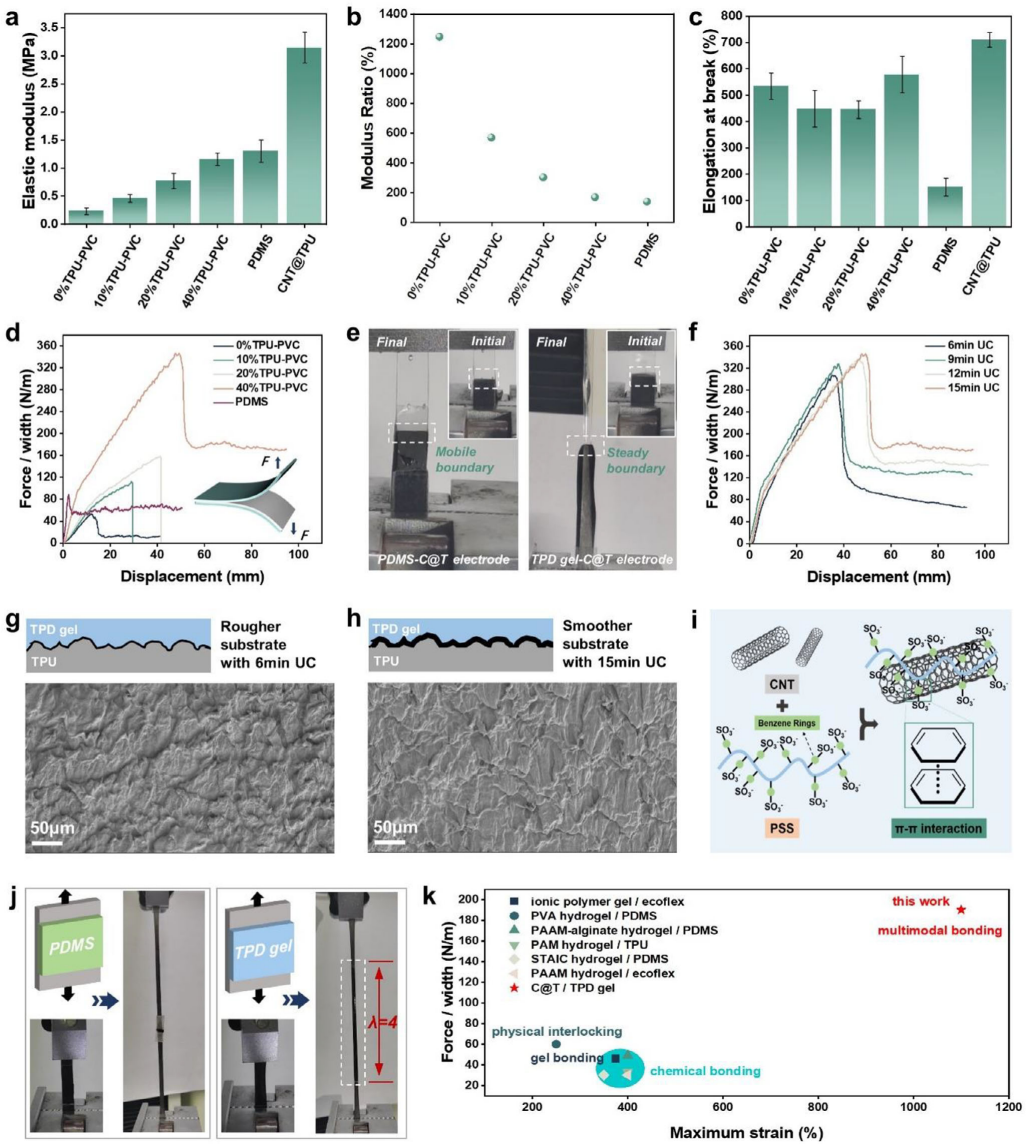
Characterization of the interface between C@T electrode and TPD gel. a) Elastic modulus of the TPD gel, PDMS, and C@T electrode. b) Calculated modulus ratio between C@T electrode and TPD gel. c) Elongation at break of the TPD gel, PDMS, and C@T electrode. d) Force–displacement curves of interfaces. Interfacial toughness was calculated using the average value of force/width at the plateau. e) Photographs of C@T‐PDMS interface and C@T‐TPD gel interface at initial and final states. f) Force–displacement curves of interfaces between C@T electrode layers subjected to different ultrasonic cavitation treatment times and the TPD gel. Surface SEM images of TPD gel peeled from C@T electrode UC treatment for g) 6 min, and h) 15 min. The scale bars were 50 µm. i) The molecular interaction mechanism of PSS/CNT. j) The high robustness of the interface under uniaxial stretches. k) Comparative analysis of the value of force/width and maximum strain in relation to previous TENG.

To investigate the interfacial properties of the C@T‐TPD gel, we first focused on validating the contribution of low modulus mismatch and interfacial strain compatibility to the enhanced interfacial stability. The customizable stretchability of the TPD gel‐based triboelectric layer was achieved by plasticizing the PVC‐DBA gel with TPU. To characterize the flexibility of the TPD gel, tensile mechanical tests were conducted. As shown in **Figure** **2**a, the elastic modulus of the TPD gel increased from 0.23 to 1.16 MPa with the addition of TPU. This improvement can be attributed to the fact that while a high content of DBA plasticizer occupies the solid space within the physically cross‐linked polymer network, thereby thinning the network and reducing mechanical properties, the subsequent incorporation of TP counteracts this effect through its inherent elasticity. Interestingly, the elastic modulus of the 40% TPU‐PVC gel closely matches that of PDMS, a commonly used triboelectric material. The modulus ratio serves as an effective parameter for quantifying interfacial mismatch in soft–soft material systems, particularly when their moduli are comparable. Based on the measured elastic moduli, the modulus ratio between the C@T electrode and the TPD gel was calculated, confirming low modulus mismatch in this system, as depicted in Figure 2b. The moduli of the 40% TPU‐PVC gel and the C@T electrode differ by less than a factor of two, fundamentally suppressing strain energy accumulation. With the significant reduction in modulus mismatch, the dominant factors influencing interfacial delamination become strain compatibility and interfacial adhesion. If there is a discrepancy in the strain tolerance between the electrode and triboelectric layers, fracture of the less compliant layer under external force or deformation can lead to stress concentration at the interface due to strain incompatibility, ultimately triggering interfacial peeling. Figure 2c shows the elongation at break for different material layers. The addition of a small amount of TPU slightly reduced the elongation of the PVC‐DBA gel, whereas a 40% TPU loading increased it. The pristine PVC‐DBA gel possesses a physically cross‐linked polymer network. When a small amount of TPU is introduced, TPU molecules disperse within this network and may form hydrogen bonds with PVC‐DBA, resulting in a slightly tighter network that restricts molecular chain slippage and thus reduces elongation under stretch. In contrast, a high TPU content leads to the formation of a continuous or interconnected TPU phase, which becomes the primary load‐bearing component. The elastic molecular chains of TPU enable extensive elastic deformation and recovery, allowing the material to withstand greater stretching and thereby increasing the overall elongation of the PVC‐DBA gel. Overall, the fracture elongation of the TPD gel and the C@T electrode are closely matched, demonstrating superior strain compatibility compared to PDMS elastomeric films.

To quantitatively evaluate the interfacial toughness, a series of peeling tests were conducted between the C@T electrode and commonly used PDMS as well as TPD gel triboelectric layers. A free end with a length equal to one‐third of the total gauge length was designed to serve as the initial stretching zone, while the remaining portion was used to assess the interfacial toughness. The boundary between these two sections allowed clear observation of the interfacial peeling process. The results are presented in Figure 2d. The stable peeling force measured for the 40% TPU‐PVC gel interface reached 190 N m^−1^, which is 3.2 times that of the PDMS interface (60 N m^−1^) and 13.8 times that of the PVC‐DBA interface (13 N m^−1^). Meanwhile, reproducibility tests were conducted by performing peel tests on multiple samples fabricated under the optimal conditions (40% TPU‐PVC, 15 min ultrasonic cavitation). The interfacial toughness value reported was derived from the average steady‐state peeling force obtained from these tests, as illustrated in Figure S4a (Supporting Information). Data from peel tests on samples with different thicknesses (Figure S4b, Supporting Information) reveals that increased thickness elevates the peeling stress at the interface, as predicted by fracture mechanics due to its effect on the neutral plane position. The high interfacial toughness of 190 N m^−1^ that we report is precisely what allows the device to manage this escalated stress and maintain reliable operation under strain.

During the peeling test of the PDMS interface, the interfacial toughness was insufficient to maintain strong adhesion. PDMS began to peel without significant deformation, and the boundary propagated easily along the interface, as shown in Figure 2e and Video S1 (Supporting Information). Similarly, the PVC‐DBA gel interface peeled readily under tension due to the high plasticizer content and migration issues. In contrast, after incorporating a small amount of TPU, the tensile curve closely followed that of the TPD gel itself throughout the process, and no boundary displacement was observed. This indicates that the free end of the TPD gel experienced the entire stretching process until fracture occurred at the boundary, with no peeling between the TPD gel and the C@T electrode. With further increase in TPU content to 40%, the 40% TPU‐PVC gel exhibited sufficient strength to sustain continuous stretching and large deformation of the free end. The boundary began to propagate along the interface only when the peeling force reached a critical value. Figure 2e and Video S2 (Supporting Information) demonstrate the ability of the TPD gel/C@T electrode interface to resist mechanical separation.

From the perspective of adhesive gel bonding, after the gel is cured in situ, it acts as an adhesive that firmly bonds to the surface of the CNT‐loaded electrospun TPU fiber electrode layer. The results of peeling tests for interfaces between C@T electrode layers subjected to different ultrasonic cavitation treatment times and the TPD gel are shown in Figure 2f. Increasing the treatment time significantly enhanced the interfacial bonding strength and toughness. A treatment duration of 15 min yielded the optimal interfacial performance, with the peak peeling force and steady‐state peeling force reaching 350 and 190 N m^−1^, respectively. This condition also exhibited the highest energy dissipation during peeling, indicating the maximum interfacial fracture energy, Γ. To further clarify the formation mechanism of the strongly bonded interface, microstructural characterization was performed on TPD gel samples peeled from electrode layers treated with 6 min and 15 min ultrasonic cavitation. As shown in Figure 2g and Figure S5a (Supporting Information), the gel peeled from the rougher substrate treated for 6 min exhibited continuous ridge‐like protrusions forming a parallel groove network. This ridge/groove structure suggests a micron‐scale periodic roughness on the substrate. During curing, the TPD gel filled these recesses, and after peeling, a “negative” morphology remained. The energy consumed during peeling was attributed to the mechanical disengagement of the gel from the protruding structures. In contrast, the gel peeled from the smoother substrate treated for 15 min (Figure 2h and Figure S5b, Supporting Information) showed mainly lamellar tearing patterns with localized curling and an absence of distinct ridge/groove features, though isolated pits were observed. The uniform tearing morphology suggests that intermolecular forces, such as van der Waals interactions and hydrogen bonding, dominated the interfacial adhesion. During ultrasonic cavitation treatment, poly(sodium styrene sulfonate) (PSS), a typical polyelectrolyte, facilitates the dispersion and adhesion of CNT through its hydrophobic long alkyl chains and benzene rings, which engage in hydrophobic and π–π interactions with the CNT, while its hydrophilic sulfonate groups form hydrogen bonds with water.^[^ ^35^ ^]^ This effect likely promotes the dispersion of CNT and their attachment to the substrate, as illustrated in Figure 2i.^[^ ^36^ ^]^ Furthermore, CNT “rootlets” bridge the interface, forming a nano‐mechanical interlock with the gel layer, thereby increasing the energy required for crack propagation.

In addition to peeling tests, the high robustness of the interface between the triboelectric layer and the electrode layer was further demonstrated under other deformation modes.^[^ ^37^ ^]^ For instance, the layered structure formed by curing TPD gel on C@T electrode fibers exhibited no delamination even when stretched to four times its original length (Figure 2j and Video S3, Supporting Information). In contrast, PDMS adhered to C@T electrode fibers detached from the electrode layer under tensile deformation (Figure 2j and Video S4, Supporting Information). These results indicate that TPU not only enables the TPD gel to suppress DBA migration, but also contributes to high interfacial toughness between the triboelectric and electrode layers. This is achieved through the polar groups in its hard segments and the penetrative flexibility of its soft segments, facilitating mechanisms such as wetting, diffusion, mechanical interlocking, and optionally, chemical bonding. In Figure 2k and Table S1 (Supporting Information), we compare our results with existing works in this field.^[^ ^21^, ^22^, ^24^, ^25^, ^26^, ^28^ ^]^ Compared to conventional gel electrode/PDMS interfaces and single⁠‐⁠strategy interfacial toughening approaches, the outstanding comprehensive performance of the C@T/TPD interface is particularly remarkable. Traditional PDMS and Ecoflex triboelectric layers often face challenges in maintaining mechanical stability under large strains. In contrast, the C@T⁠‐⁠TPD gel demonstrates superior stretchability (exceeding 1110%) and high interfacial toughness (190 N m^−1^) when compared to existing TENG devices.

An accelerated aging test was conducted to evaluate the migration trend of the plasticizer DBA and the efficacy of TPU in suppressing it. Based on the principle that high temperature accelerates the migration and leaching of small‐molecule plasticizers, pure PVC‐DBA gel and 40% TPU‐PVC gel samples were subjected to aging at 60 °C for 144 h. A comparative analysis of the weight loss rates revealed that TPU significantly enhanced the retention of DBA, with the 40% TPU‐PVC sample showing a weight loss of only 10% (Figure S6a, Supporting Information). Furthermore, peel tests were conducted on the aged C@T‐TPD samples. As shown in Figure S6b (Supporting Information), the interfacial toughness of the pure PVC‐DBA gel with the electrode decreased dramatically due to the interfacial weakening caused by plasticizer migration. In contrast, the 40% TPU‐PVC gel sample maintained a high level of interfacial adhesion. Meanwhile, we fixed the C@T‐TPD gel on the tensile testing machine and conducted a continuous 1000 cycles of 100% strain cyclic tensile tests, as shown in Figure S6c (Supporting Information). After the tensile tests, the samples were subjected to peel tests to quantitatively measure the change in interface toughness. The results showed that the steady‐state peel force remained consistent before and after the tensile process, as shown in Figure S6d (Supporting Information).

### 2.3 Mechanical Properties and Sensing Performance of the C@T‐TPD Gel Strain Sensor

**Figure 3:**
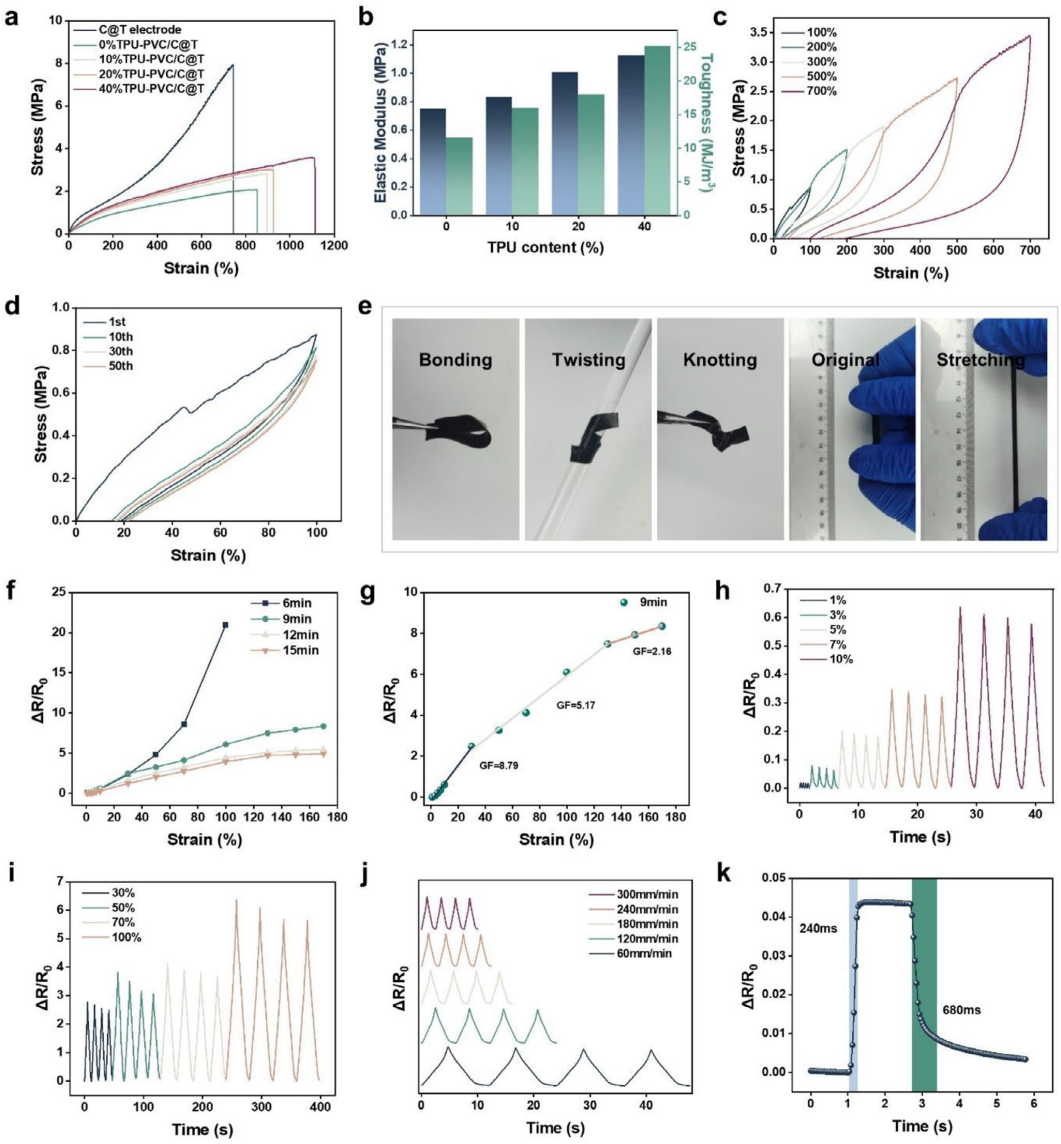
Mechanical properties and sensing performance of the C@T‐TPD gel strain sensor. a) Tensile stress–strain curves of C@T electrode and C@T‐TPD gels with different contents of TPU. b) Toughness and modulus of C@T‐TPD gels. c) Tensile loading–unloading curves of the C@T‐TPD gel contain 40% TPU at a strain of 100%, 200%, 300%, 500%, and 700%. d) Cyclic stress–strain curves of the C@T‐TPD gel contain 40% TPU. e) Optical images of C@T‐TPD gel bending, twisting, knotting, and stretching. f) Response of resistance change to tensile strain for the strain sensors composed of fiber mats decorated with different times (6, 9, 12, 15 min) of UC treatment. g) Δ*R*/*R*_0_ upon the applied strains for strain sensor with 9 min UC treatment. h) Dynamic response in the small range of strain (1%–10%). i) Dynamic response in the large scale of strain (30%–100%). j) Δ*R*/*R*_0_ of strain sensor at diverse frequencies under 10% strain. k) Response and recovery time of strain sensor.

To understand the effect of TPU content in the TPD gel on the mechanical properties of the C@T‐TPD gel, tensile tests were conducted. As shown in **Figure** **3**a, by varying the TPU content, the elongation at break of the C@T‐TPD gel could be tuned from 850% to 1110%, and the fracture stress could be adjusted from 2 to 3.5 MPa. Correspondingly, both the elastic modulus and toughness of the C@T‐TPD gel increased with higher TPU content (Figure 3b). Interestingly, when the TPD gel was combined with the C@T electrode, the resulting C@T‐TPD composite gel showed a significant increase in elongation at break compared to the individual C@T electrode and TPD gel, while the fracture stress decreased markedly. Meanwhile, the overall modulus of the composite fell between 0.75 and 1.12 MPa, which is considerably lower than the elastic modulus of the pure C@T electrode (3.15 MPa). The alterations in the mechanical properties of the C@T‐TPD gel can be attributed to a soft‐matrix‐dominated mechanism. During stretching, the soft TPD gel matrix undergoes large deformation first, leading to the composite exhibiting a higher elongation at break (850%–1110%) than both the pure C@T electrode (700%) and the pure TPD gel (600%). The low strength of the soft matrix also reduces the overall strength of the composite, resulting in a fracture stress significantly lower than that of the pure C@T electrode. Similarly, the low modulus of the soft matrix governs the initial mechanical response of the composite, causing the overall elastic modulus (0.75–1.12 MPa) to be much lower than that of the pure C@T electrode (3.15 MPa).

To gain deeper insight into the mechanical properties of the C@T‐TPD composite gel, we investigated its behavior during tensile loading–unloading cycles to calculate the corresponding energy dissipation (Figure 3c). As the strain increased from 100% to 700%, the dissipated energy rose from 0.24 to 6.46 MJ m^−^^3^ (Figure S7, Supporting Information). When the strain was fixed at 100%, the largest hysteresis loop was observed in the first cycle. In subsequent cycles, the energy dissipated per cycle decreased significantly and gradually stabilized (Figure 3d). This behavior is attributed to the reorganization of the internal network and stress redistribution within the C@T‐TPD composite gel under cyclic loading. As the number of cycles increased, the internal energy dissipation and damage accumulation reached a relatively balanced state. Ductility is another crucial parameter for evaluating mechanical performance. The C@T‐TPD composite exhibits excellent ductility, enabling it to withstand considerable deformation. Furthermore, as shown in Figure 3e, the C@T‐TPD demonstrates outstanding flexibility, capable of conforming to and recovering from deformations of various shapes.

The presence of a conductive fiber network within the C@T‐TPD composite gel imparts strain‐sensing capabilities to the conductive material. The loading amount of CNT significantly influences the initial electrical resistance of the flexible strain sensor. As shown in Figure 3f, strain sensors with conductive networks of different CNT loadings exhibit distinct sensing behaviors. The sensor based on C@T‐TPD subjected to 6 min of ultrasonic cavitation treatment contains a relatively low CNT loading, resulting in poor stretchability. Consequently, its sensing functionality is lost when the strain exceeds 100%. With longer ultrasonic cavitation treatment times of 9 to 15 min, higher CNT loading reduces the initial resistance of the conductive layer, facilitating both high sensitivity and a broad sensing range. Among these, the sensor treated for 9 min demonstrates superior performance across a wide strain range (0–170%) and a more pronounced change in resistance compared to other samples. The electromechanical performance of this sensor was further evaluated, and the relative resistance change as a function of strain is presented in Figure 3g. The tensile process can be divided into three distinct strain regions based on the resistive response: 0–30%, 30–130%, and 130–170%, corresponding to gauge factors (GF) of 8.79, 5.17, and 2.12, respectively. Since daily human motion involves varying amplitudes and frequencies, tensile tests were conducted to evaluate the sensor's performance under realistic conditions, which is essential for practical applications. The relative resistance changes under small strains (1–10%) and large strains (30–100%) are shown in Figure 3h,i, respectively. The resistance increases systematically with applied strain. The sensor's reversibility stems from the excellent elastic properties of the gel film, which allows it to gradually return to its initial state even after being stretched to 200%. Figure 3j shows the relative resistance change of the sensor under 10% strain at different stretching rates. As the rate increased from 60 to 300 mm min^−1^, the sensor exhibited consistent responses with stable signal amplitude, indicating that the strain rate has negligible influence on its sensing performance. Additionally, the sensor showed a rapid response time of 240 ms when subjected to 1% strain (Figure 3k). The low initial resistance provided by the CNT network contributes to high sensitivity, while stable electrical contacts among CNT ensure consistent conductive pathways. To assess the sensor's fatigue resistance, dynamic durability tests were performed under 30% cyclic strain. As indicated in Figure S8 (Supporting Information), the sensor maintains stable performance with minimal fluctuation even after 1000 stretching cycles. Enlarged views of the initial and final cycles confirm highly reproducible resistance changes in every cycle, demonstrating outstanding fatigue resistance and sensing stability.

### 2.4 Output Performances of C@T‐TPD Gel‐Based TENG with Stretchable and Robust Interfacial

**Figure 4:**
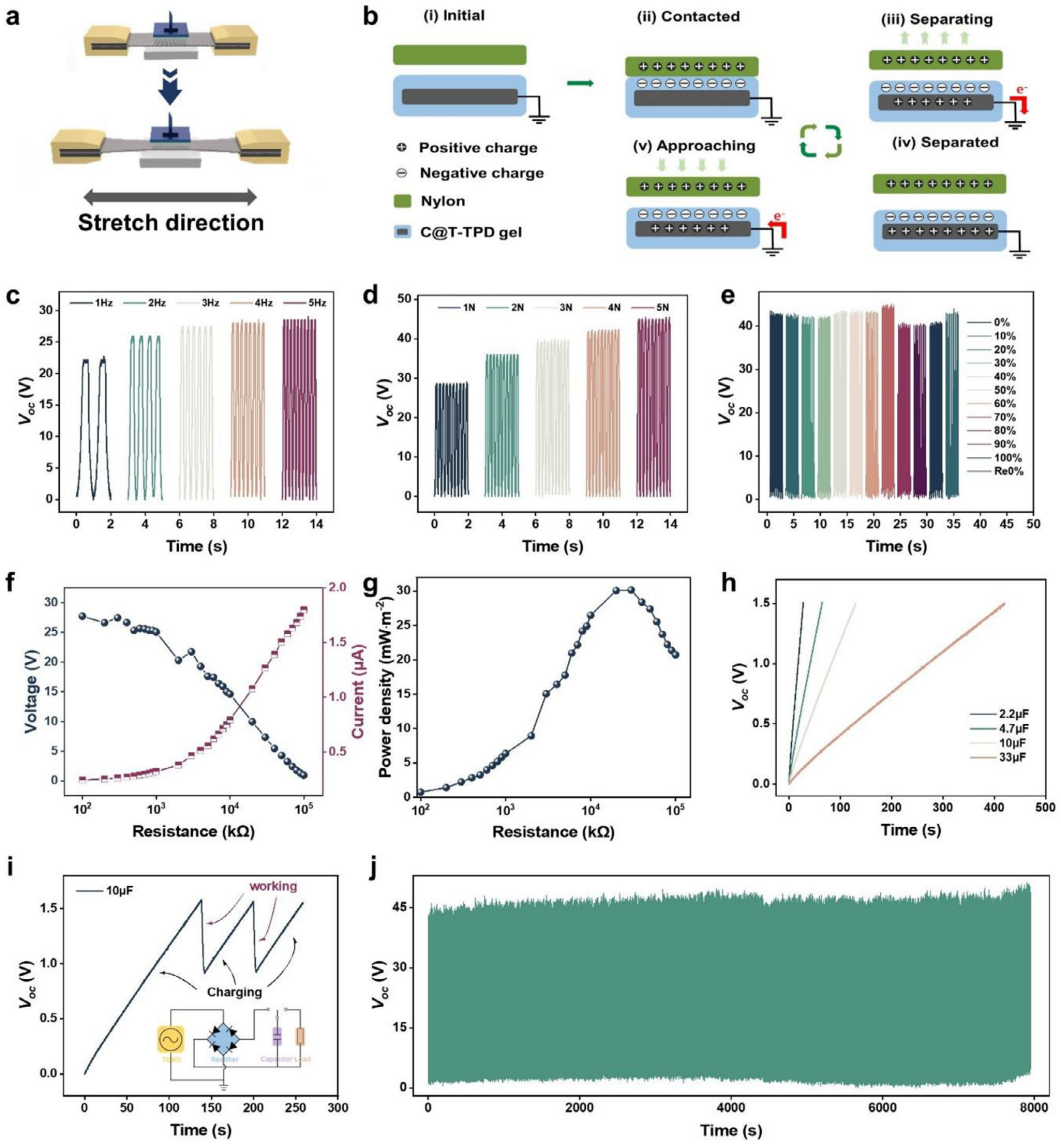
Self‐generation capacity of the C@T‐TPD gel‐based TENG. a) Test process of the TENG. b) Schematic diagram illustrates the working mechanism of TENG. Voltage output c) under different contact–separation frequencies (1–5 Hz), d) under different forces (1–5 N) and e) under different strains (10%–100%). Relationship of f) the output voltage, output current and g) power density as a function of different external loading resistors of 100 kΩ–100 GΩ. h) Analysis of the charging performance for different capacities. i) Electric circuit and charge–discharge process of the charging system using TENG as energy supply for powering up electronics. j) Performance durability during 30 000 contact–separation cycles.

Having established the ability of the C@T‐TPD gel interface to form a robust bond between the triboelectric and electrode layers, we further utilized this concept to fabricate TENG that combine flexibility and stretchability with high reliability and mechanical robustness, as illustrated in **Figure** **4**a. The gel‐based triboelectric device was designed to operate in single‐electrode mode, with its working mechanism depicted in Figure 4b. Specifically, in the initial state (I), no charge is present on the surface of either triboelectric material. When nylon (a positive triboelectric material) comes into full contact with the TPD gel (a negative triboelectric material), opposite yet equal amounts of charge are generated on their surfaces due to the triboelectric effect, with no current flow occurring at this stage (II). As the nylon separates from the TPD gel, the negative charges on the gel induce positive charges on the C@T electrode, resulting in a flow of current through the external circuit (III). When the nylon is completely removed and remains at a sufficient distance, the negative charges on the gel are fully screened by the induced positive charges on the C@T electrode, and electron flow ceases (IV). Finally, as the nylon approaches the TPD layer again, the previous electrostatic balance is disrupted, causing electrons to flow in the opposite direction through the external circuit to reestablish charge equilibrium (V). The repeated pressing and releasing motions generate alternating electrical signals, which manifest as a series of positive and negative electrical pulses.

The electrical output of the C@T‐TPD gel‐based TENG under varying frequencies, pressures, and strains is shown in Figure 4c–e. Notably, in Figure 4c, the output signals of the TENG are clearly distinguishable within the frequency range of 1 to 5 Hz. The output voltage increases with higher frequencies, which can be attributed to the increased contact–separation speed and the associated dynamic force at higher frequencies, leading to more efficient charge transfer and induction. As illustrated in Figure 4d, the output voltage also rises with increasing pressure. The voltage output across strains ranging from 0% to 100% is presented in Figure 4e. A slight decrease in voltage is observed when the strain exceeds 80%, but the output recovers to its original level once the strain is released. The corresponding short‐circuit current and transferred charge under different test parameters are provided in Figure S9 (Supporting Information). TENG output tests across a wide strain range were performed to directly assess the device's performance limits (Figure S10, Supporting Information). The results indicate a gradual decay in the output signal with increasing strain, although a measurable output persisted at high strains. Ultimately, a catastrophic failure was observed beyond 500% strain, attributable to a pronounced deterioration in the robustness of the CNT conductive network. Our previous data demonstrated device stability for strains applicable to the human body (≤100%). This demonstrates that the device is far from its failure point at this strain, providing strong preliminary evidence for its capability to withstand large deformations. An analysis of the influence of gel thickness was investigated (see Figure S10, Supporting Information). The observed performance degradation with excessive thickness is attributed to two main factors: first, the increased thickness reduces the effective capacitance, limiting current and charge output; second, the thick layer impedes efficient charge induction, as a significant portion of the charges become dissipated or shielded before reaching the back electrode, resulting in reduced voltage.

Furthermore, the relationship between output voltage, current, power, and external load resistance was systematically investigated by varying the external resistor (Figure 4f). Over a resistance range from 100 kΩ to 100 MΩ, the output voltage rises rapidly with increasing resistance and eventually reaches saturation. A maximum power density of 31 mW m^−^^2^ was achieved at a load resistance of 20 MΩ. To explore its potential as a self‐powered device, the C@T‐TPD gel‐based TENG was connected to a rectification circuit to evaluate its capability to power electronic devices (Figure 4h,i). Commercial capacitors with capacitances of 2.2, 4.7, 10, and 33 µF were used to assess the charging performance. As the capacitance increases, the charging rate decreases. The time required to reach 1.5 V was 27 s for the 2.2 µF capacitor, 65 s for the 4.7 µF capacitor, and 125 s for the 10 µF capacitor. In addition, the alternating current generated by the TENG was converted to direct current via a rectifying bridge to charge a 10 µF capacitor, which successfully powered an electronic watch. Moreover, the optimized output performance in Figure 4f–h, the input force is 4 N under the frequency of 4 Hz. Since stability is a critical factor for wearable electronics in practical applications, the long‐term signal stability of the TENG was also evaluated (Figure 4j). The results demonstrate the reliable power supply capability of the TENG and its significant potential as a self‐sufficient energy source for wearable sensors.

### 2.5 Multifunctional Application of C@T‐TPD Gel‐Based Strain Sensor and TENG

**Figure 5:**
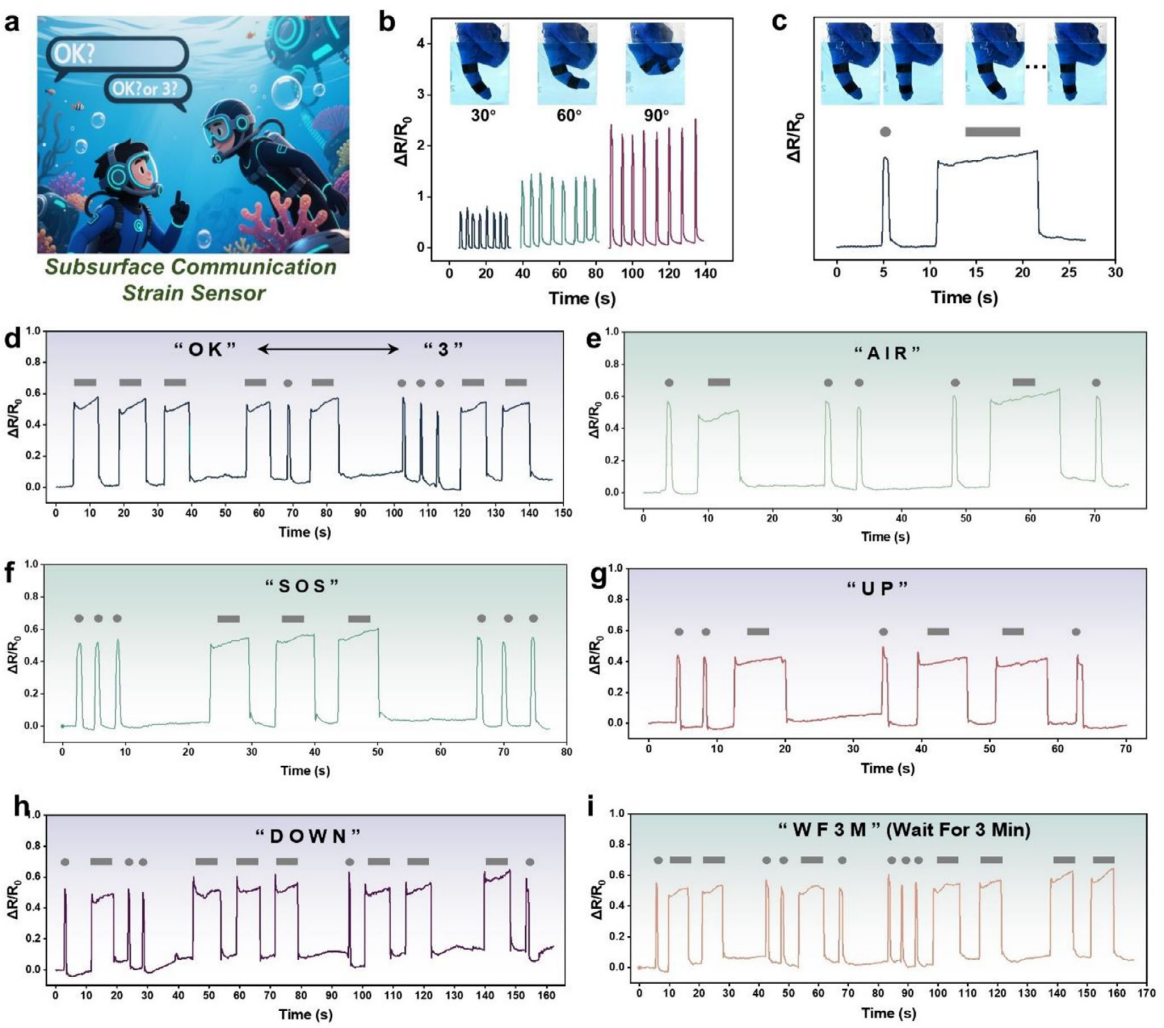
Waterproof strain sensor based on C@T‐TPD gel. a) Dilemmas encountered by divers in gesture communication. b) Electrical signal reflection of underwater finger bending. c) The definition of "dot" and "dash" signals depending on the deformation time. d–i) The raw signals of different Morse codes by controlling the deformation time of the Waterproof strain sensor.

In the C@T‐TPD gel system, the TPD layer functions as a negatively charged component and encapsulates the C@T fiber electrode. This encapsulation not only enhances the stability of the sensor but also imparts water‐resistant properties. Conventional communication devices often perform poorly underwater, typically forcing divers to rely on hand signals for communication. However, factors such as poor visibility and undersea environmental pressures can complicate the accurate interpretation of these signals. For example, the gestures for “OK” and “3” are easily confused underwater, leading to ineffective communication (**Figure** **5**a). To address this challenge, a waterproof strain sensor based on the C@T‐TPD gel was developed. As shown in Figure S11 (Supporting Information), the sensor was affixed to a finger joint using waterproof tape and immersed in a beaker of water to simulate an underwater environment. Figure 5b illustrates the variation in electrical signals corresponding to different finger bending angles underwater, demonstrating the accuracy and sensitivity of the sensor. Accordingly, a Morse code‐based signaling strategy linked to finger gestures was established (Figure 5c). This system allows clear differentiation between easily confused signals such as “OK” and “3” (Figure 5d). Since oxygen supply is critical for diver safety, the sensor was used to accurately transmit the message “AIR”, which can be interpreted as a request for oxygen (Figure 5e). Figure 5f–h demonstrates the successful transmission of messages such as “SOS,” “UP,” and “DOWN,” which correspond to situations like emergencies and ascent/descent instructions. The requirement for a diver to remain underwater for 3 min before resurfacing represents an essential safety protocol; the transmission of the signal “WF3M”, as shown in Figure 5i, offers vital guidance during this phase. Thus, the capability of this waterproof strain sensor to facilitate underwater communication is highly valuable. It provides a reliable means of conveying information, enhancing both the safety and efficiency of diving operations.

**Figure 6:**
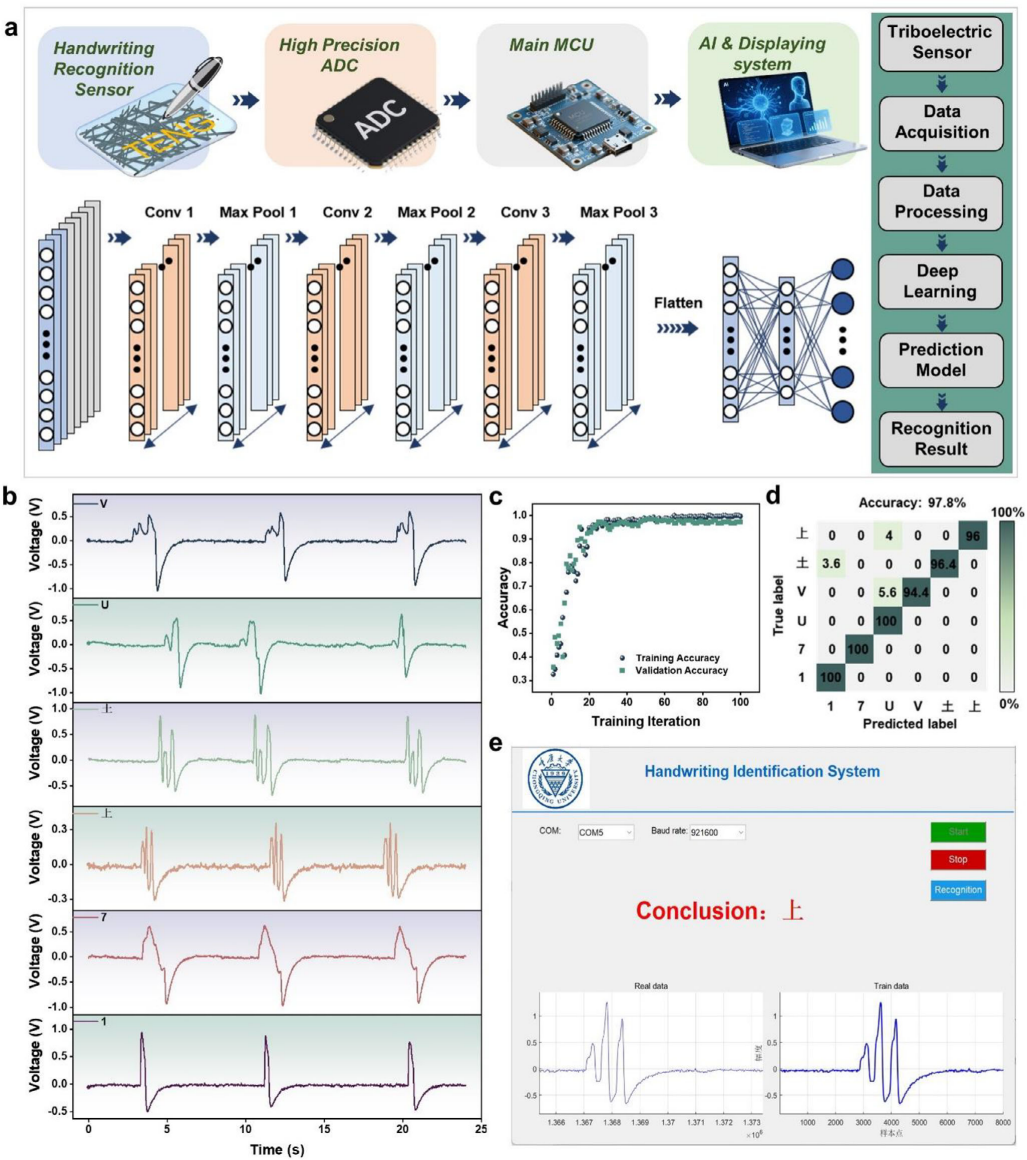
Handwritten recognition based on C@T‐TPD gel‐based TENG with deep learning. a) The flowchart of the system and detailed structure of the 1D‐CNN model. b) Voltage signals of handwriting English character, Chinese character, and numerical character. c) The relationship between identification accuracy and the number of training iteration. d) Predicted confusion matrix of handwritten recognition of 6 different characters. e) Recognition results of Chinese character “上” (up).

Delamination resistance is essential for self‐powered handwritten recognition devices to withstand dynamic mechanical loads during writing and maintain the structural integrity of the triboelectric‐electrode interface. Without such resistance, structural failure would occur before the device could function, and accurate alignment between the triboelectric signals and pen‐tip motion would be compromised, leading to signal distortion and unreliable handwritten recognition. In summary, robust dynamic triboelectric signals are required for handwritten recognition, which in turn depends on strong adhesion between the triboelectric and electrode layers to ensure both mechanical force transfer and stable electrical contact. Delamination resistance is therefore critical to maintaining this interfacial cohesion. Using C@T‐TPD gel‐based TENG along with a nylon‐tipped pen, we developed a delamination‐resistant handwritten recognition sensor. This sensor was integrated with a signal acquisition circuit (Figure S12, Supporting Information) and a deep learning algorithm to construct an intelligent handwritten recognition system, as illustrated in **Figure** **6**a.

The convolutional neural network (CNN) is a deep learning model that employs convolutional operations to extract local features and capture spatial structures from data. By utilizing convolutional layers for feature extraction, pooling layers to reduce feature dimensionality and improve computational efficiency, and fully connected layers for feature integration and output, its parameter‐sharing mechanism and sparse connectivity significantly reduce computational complexity, making it well suited for character recognition applications. The CNN algorithm was trained to classify and recognize English letters, digits, and Chinese characters. A total of 600 samples were collected, with 100 samples per character. The dataset was split into 70% for training and 30% for testing to ensure a balanced evaluation of the model. Distinct triboelectric signals corresponding to highly similar written characters—such as V and U, “土” (earth) and “上” (up), 7 and 1—are shown in Figure 6b. By extracting multi‐dimensional features from the output waveforms of the TENG and training the CNN model over 100 epochs, the number of training iterations was studied and an optimal value was determined. As shown in Figure 6c, the accuracy converges after 20 epochs and remains stable throughout 100 training cycles, demonstrating the model's high recognition performance and strong robustness. The character recognition system accurately identified six different types of characters. A confusion matrix (Figure 6d) shows a recognition accuracy of 97.8%, and the real‐time recognition results were displayed on a computer terminal (Figure 6e). This high level of accuracy confirms the reliability of the C@T‐TPD gel‐based TENG in character recognition tasks.

## 3 Conclusion

This study successfully designed and fabricated a high‐performance, stretchable TENG based on a homologous polymer pairing strategy. By constructing both the triboelectric layer (TPD gel) and the electrode layer (C@T) on a consistent polyurethane matrix, modulus matching (modulus ratio < 2) and excellent strain compatibility were achieved, fundamentally suppressing interfacial stress accumulation and delamination. Through ultrasonic cavitation treatment, the interfacial toughness was significantly enhanced to 190 N m^−1^, which is 3.2 times that of conventional PDMS‐based interfaces, while structural integrity was maintained even under 400% stretching and complex deformations. The C@T‐TPD gel‐based TENG maintains high triboelectric charge density while also exhibiting strain‐insensitive conductivity (stable resistance under 200% strain), extensibility exceeding 100%, and excellent fatigue resistance. In practical applications, the device demonstrates stable energy output (maximum power density of 31 mW m^−^^2^), outstanding stretchability (stable output at 100% strain), exceptional cyclic durability, and promising performance in waterproof strain sensing and underwater communication. Leveraging the delamination‐resistant behavior of the triboelectric sensor integrated with a deep learning algorithm, an intelligent character recognition system was constructed, achieving high‐precision recognition (97.8% accuracy). These results highlight its potential applications in flexible energy sources, wearable health monitoring, and human–machine interaction. This work places our findings within the context of advancing stretchable TENG technologies, including those based on carbon networks and gels, and identifies the in depth analysis of Poisson's ratio effects under complex strain as a key objective for future research. While the present validation is confined to a TPU homologous system, the underlying paradigm of “modulus matching and interfacial enhancement” is expected to be transferable to other polymer families with similar chemical affinities (e.g., polyacrylates, silicones, or SEBS). Subsequent research will be directed toward validating this potential across a wider array of material systems.

## Conflict of Interest

The authors declare no conflict of interest.

## Supporting information



Supporting Information

Supplemental Video 1

Supplemental Video 2

Supplemental Video 3

Supplemental Video 4

## Data Availability

The data that support the findings of this study are available from the corresponding author upon reasonable request.
