# Supplementary material for: Modulus Matching and Interface Enhancement: A Synergistic Strategy for Antidelamination High‐Performance Stretchable Triboelectric Nanogenerator
Source: Adv Sci (Weinh). 2025 Nov 19;13(6):e18027. doi: 10.1002/advs.202518027 (PMC12866878; doi:10.1002/advs.202518027)
Supplement: Supplementary file 1 — Supporting Information [file ADVS-13-e18027-s002.docx]

Supporting Information

**Modulus Matching and Interface Enhancement: A Synergistic Strategy for Anti-Delamination High-Performance Stretchable Triboelectric Nanogenerator**

*Shiwei Xu^1^, Pengfan Wu^1^, Feng Qin^1^, Endian Cui^1^, Zhongyong Mo^1^, Hengyu Guo^2^, Xiaojing Mu^1^*, Hua Yu^1^**

S. Xu^1^, P. Wu^1^, F. Qin^1^, E. Cui^1^, Z. Mo^1^, X. Mu^1^*, H. Yu^1^*

^1^Key Laboratory of Optoelectronic Technology & Systems Ministry of Education, International R&D Center of Micro-Nano Systems and New Materials Technology, Chongqing University, Chongqing 400044, China.

H. Guo^2^

^2^State Key Laboratory of Mechanical Transmission, College of Mechanical and Vehicle Engineering, Chongqing University, Chongqing 400044, China

E-mail: mxjacj@cqu.edu.cn; yuhua@cqu.edu.cn.

**Experimental Section**

*Materials*: Thermoplastic polyurethane (TPU, Elastollan 1185A), Poly(sodium-p-styrenesulfonate) (PSS, Mw ~ 70,000), nylon 11 (pellets, 3 mm), Tetrahydrofuran (THF, ≥ 99.9%, AR), N, N-dimethylformamide (DMF, 99.8%, AR), Polyvinyl chloride (PVC, Mw ~ 95,000), Dibutyl adipate (DBA, ≥ 99%, AR) were purchased from Shanghai Aladdin Biochemical Technology Co. Carbon nanotube (CNT) with diameters of 20–30 nm and lengths of 10–30 μm was obtained from Time Nano Co. Ltd. All regents were used as received without further treatments.

*Preparation of TPU fibers by electrospinning*: TPU fibrous membrane was prepared by an electrostatic spinning equipment (Model ET-2535, Beijing Ucrane Technology Development Co. Ltd). Firstly, TPU solution with a concentration of 20 wt% was prepared by dissolving a certain quality of TPU particles into DMF/THF mixture solution with a volume ratio of 1:1 under stirring for 12 h at room temperature. The obtained clear TPU solution was then added into a 5 mL disposable syringe with 20-gauge stainless steel needle. After that, the TPU solution was electrospun into fibers with collector positioned at 10 cm away from the tip of spinneret, and the applied voltage and flow rate were set as 10 kV, 0.1 mL h 1, respectively. Finally, The TPU fibrous mat was peeled off from the collector and put into an oven at 60 ℃ for over 2 h to evaporate the residual solvent.

*Preparation of C@T electrode by ultrasonic cavitation (UC)*: The decoration of CNT onto electrospun TPU fibers was achieved by ultrasonic cavitation treatment. For the preparation of reaction solution, 50 mg CNT and 50 mg PSS were added to 100 mL of deionized water, and then the suspension was dispersed under ultrasonication for 2 h to reach a good homogeneity. After that, the prepared electrospun TPU mat was immersed in the CNT suspension and subjected to ultrasonication cavitation treatment in an ultrasonic horn (SCIENTZ-IID, Scientz, China) under a power of 250 W. The treatment time was ranged from 6 to 15 min. The prepared membrane was washed out with deionized water several times and dried at 60 ℃ in an oven for 24 h.

*Preparation of TPD gel:* First, the mixture of DBA and THF was stirred at room temperature for 10 min. TPU and PVC powder were added to the above mixture, which was then sonicated for 10 min. The treated mixture was stirred with a magnetic stirrer at 1500 rpm for 45 h to obtain a mixture precursor suspension. After that, a proper amount of the precursor solution was drop-cast onto the prepared C@T membrane. Finally, the precursor suspension was evaporated and cured at room temperature for approximately 72 h to obtain the TPD gel. The TPU to PVC weight ratios were 0%, 10%, 20%, and 40%. The mass ratio of PVC to DBA was kept constant at 1:2.

*Preparation of the strain sensor and the* *C@T-TPD gel-based TENG:* The strain sensor was fabricated using the as-prepared C@T-TPD gel, which was cut into rectangular-shaped membranes (3 cm × 0.5 cm) and loaded with two copper (Cu) wires to assemble into stretchable sensors. The C@T-TPD gel TENG was fabricated using the as-prepared C@T-TPD gel. The gel was cut into a 3 cm × 3 cm piece and connected to a Cu wire for electrical connection.

*Characterization:* The micromorphology of the materials was tested using JSM 6390 field emission scanning electron microscope (SEM), then the photoelectron spectroscopy test was carried out. Electrospun TPU fibers were directly deposited onto a copper grid for a few seconds and then the fibers were treated through ultrasonic cavitation treatment for TEM observation (JEM-F200, JEOL). The Raman measurements were performed at room temperature through a micro-Raman spectroscopy system (HR 800, Horiba Scientific) with a 514.5 nm laser for excitation. The FT-IR spectra were measured with the High-Resolution FTIR (Bruker Vertex 80v) by attenuated total reflection (ATR) mode in the range of 4000 – 400 cm^-1^. The strain sensing performances were tested using the testing equipment consists of Source Measure Unit (KEITHLEY 2612B) and universal testing machine (UTM2203, Suns Technology Co. Ltd.). During the testing process, the resistance of sensor was online monitored and recorded, and the relative resistance variation ΔR/R_0_ (ΔR = R-R_0_, where R and R_0_ represents the resistance of sensor in the tensile state and the initial state, respectively) was calculated to evaluate its strain sensing performance. In addition, Gauge factor (GF) is used to evaluate the sensitivity of strain sensor, which can be calculated using the following Equation: $GF=\frac{\Delta R/R_{0}}{\varepsilon}$, where ε represents the applied strain. The output performances of C@T-TPD gel TENG were measured in the vertical contact mode using a 3 cm × 3 cm. The stretchable single-electrode C@T-TPD gel TENG had an active area of 2 cm × 2 cm (contact material: nylon). The open-circuit voltage (V_OC_), the short-circuit current (I_SC_), and generated charge (Q_SC_) were measured using a system electrometer (KEITHLEY 6514).


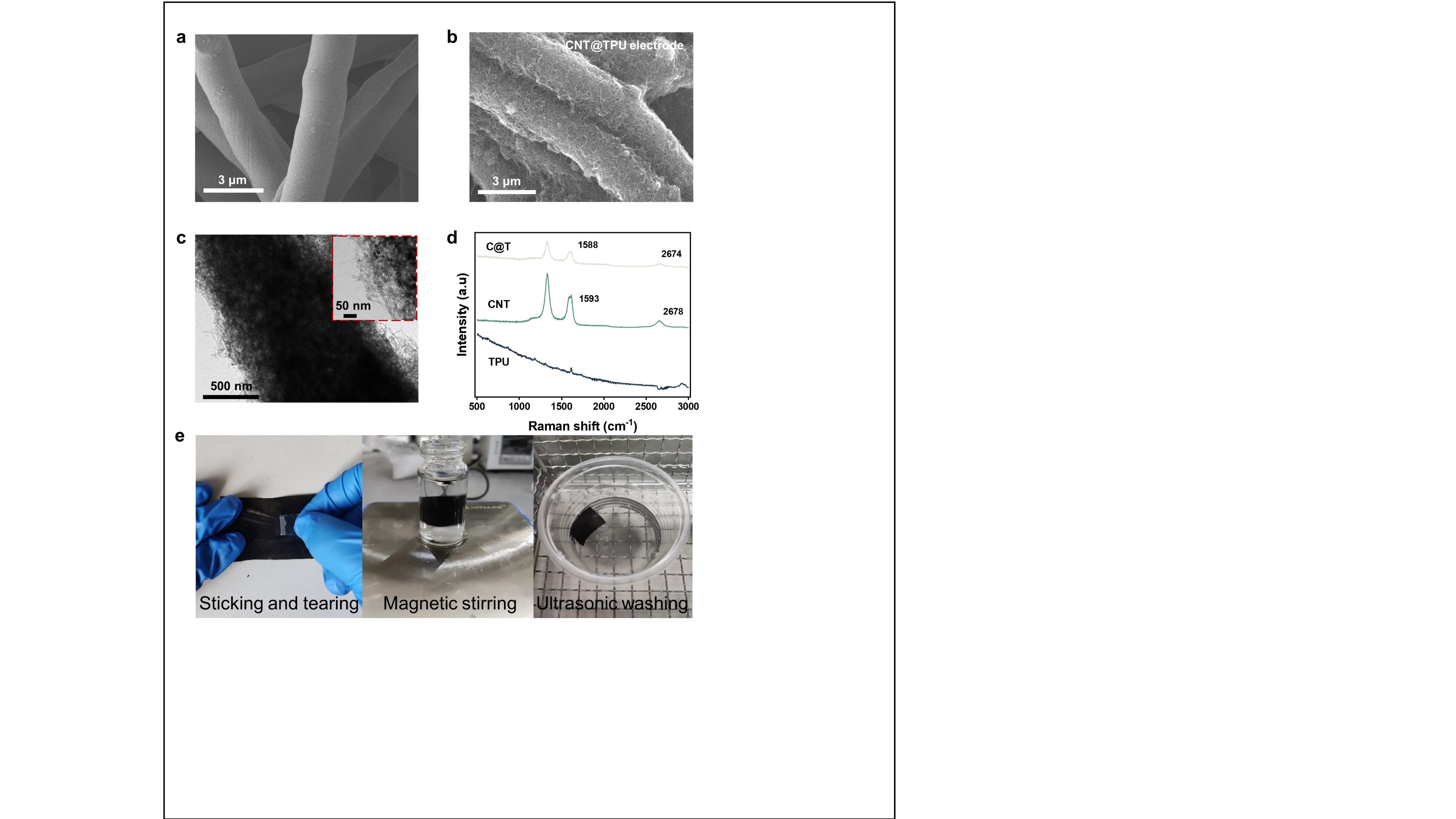


**Figure S1.** The SEM of (a) TPU and (b) C@T electrode. (c) The TEM images of the C@T electrode. (d) Raman spectra of pure TPU, CNT, and C@T electrode. (e) The outstanding durability even under repeated sticking and tearing by scotch tape, magnetic stirring in sodium chloride aqueous solution, and ultrasonic washing in deionized water.


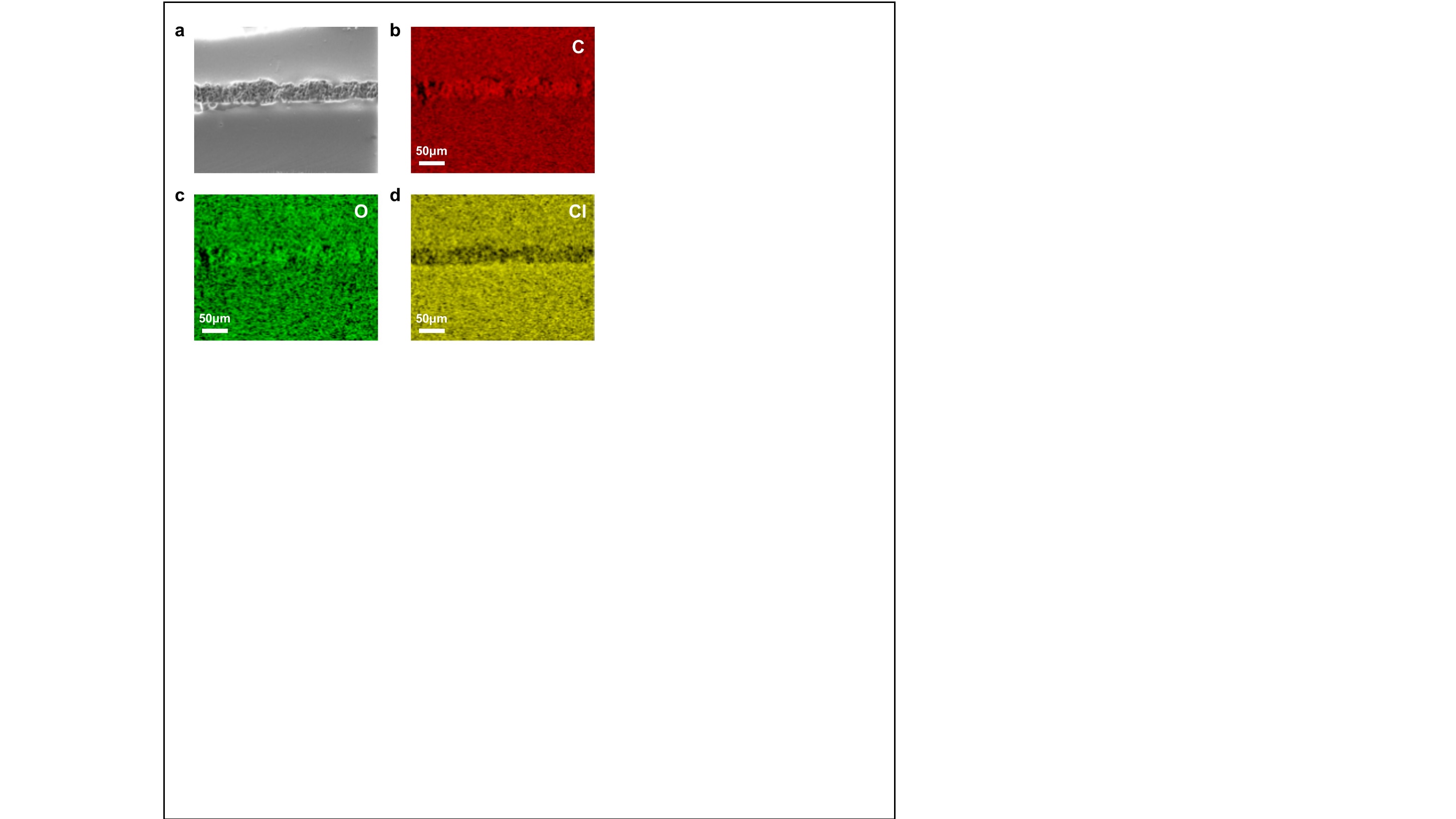


**Figure S2.** Cross-sectional SEM images and EDS elemental mapping of C@T-TPD gel.


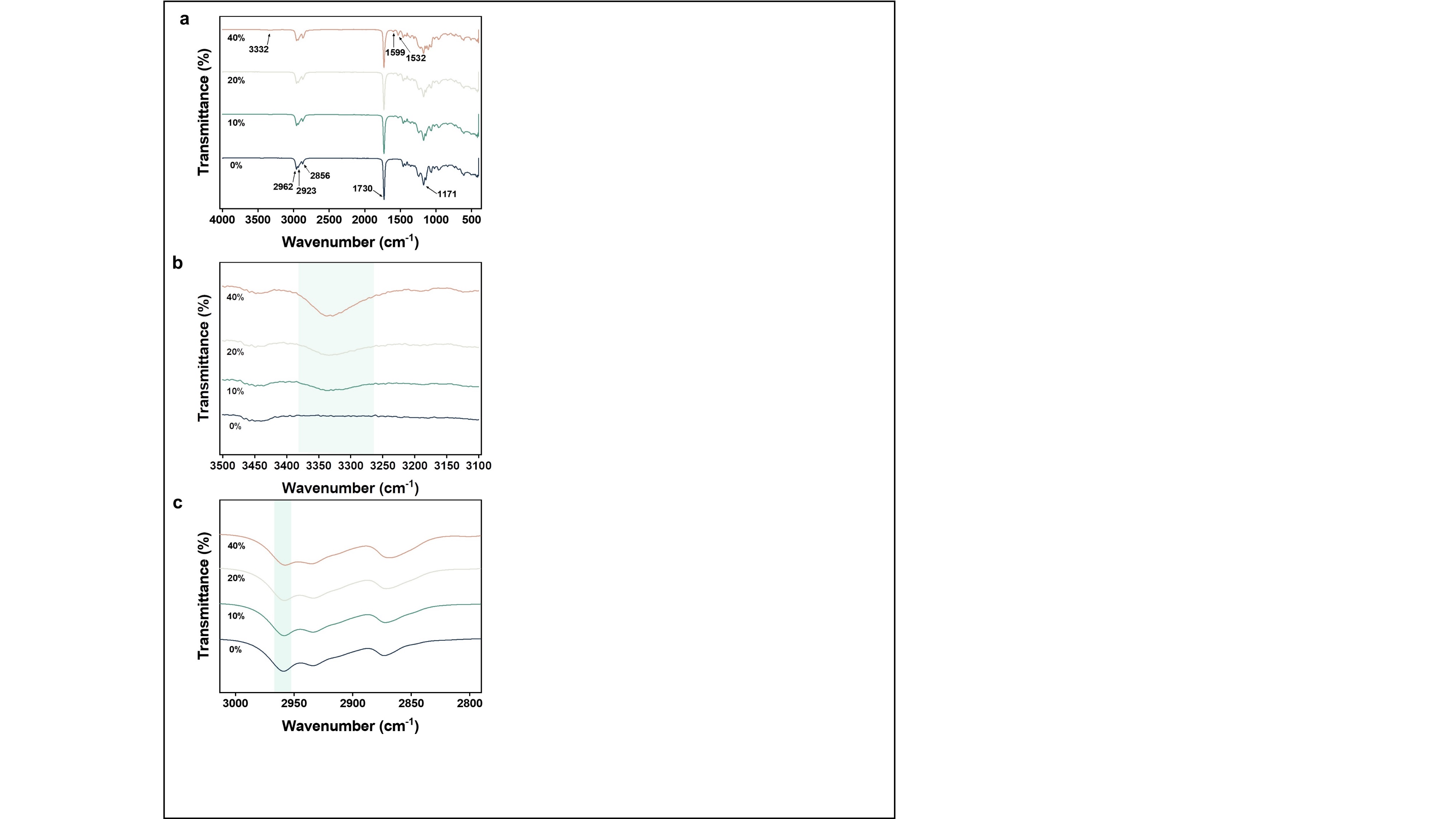


**Figure S3.** The FTIR spectra of TPU-PVC composites with different blending ratios.


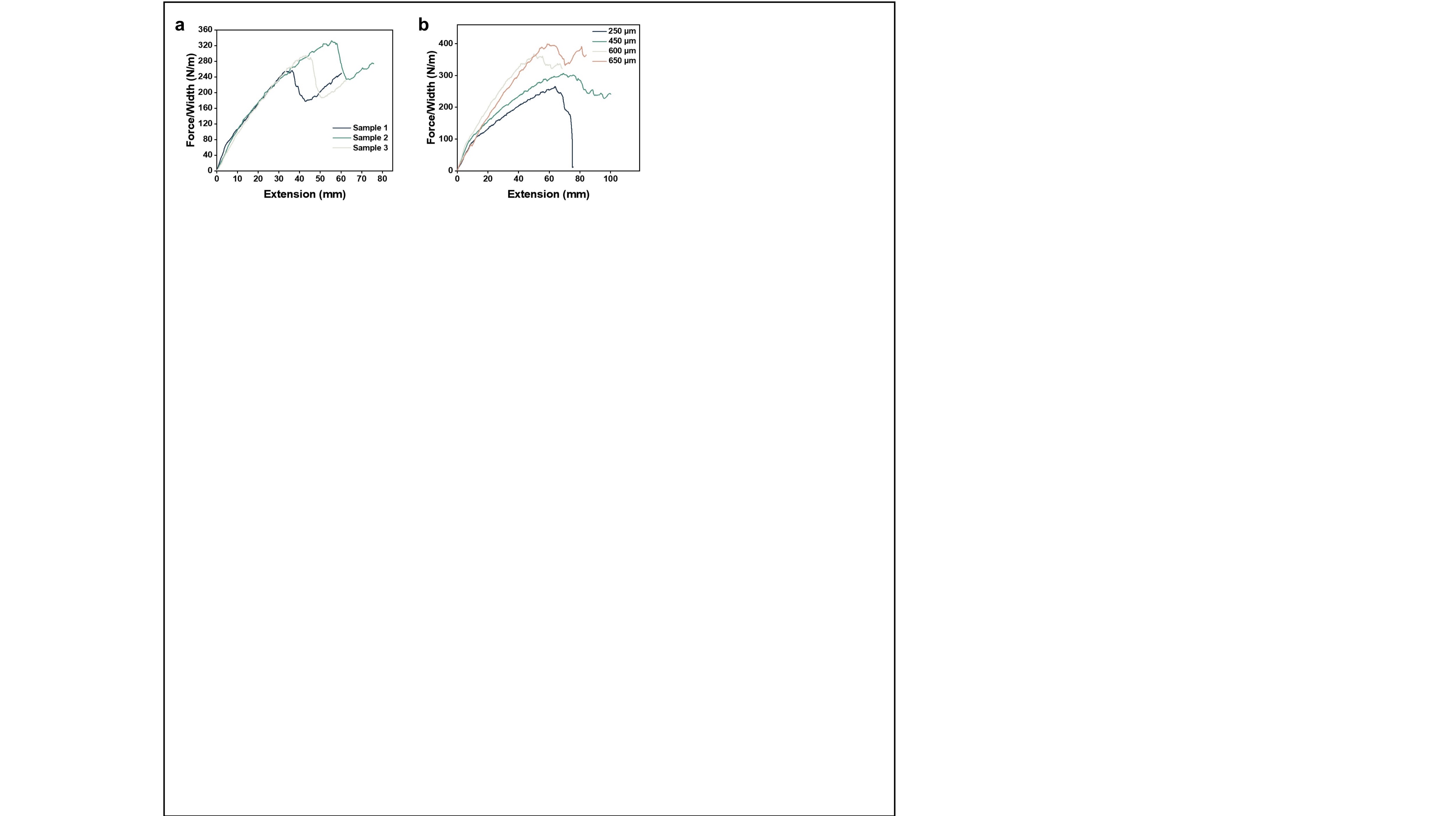


**Figure S4.** (a) Reproducibility tests of interfacial toughness value. (b) Force-extension curves of interfaces for different gel thicknesses.
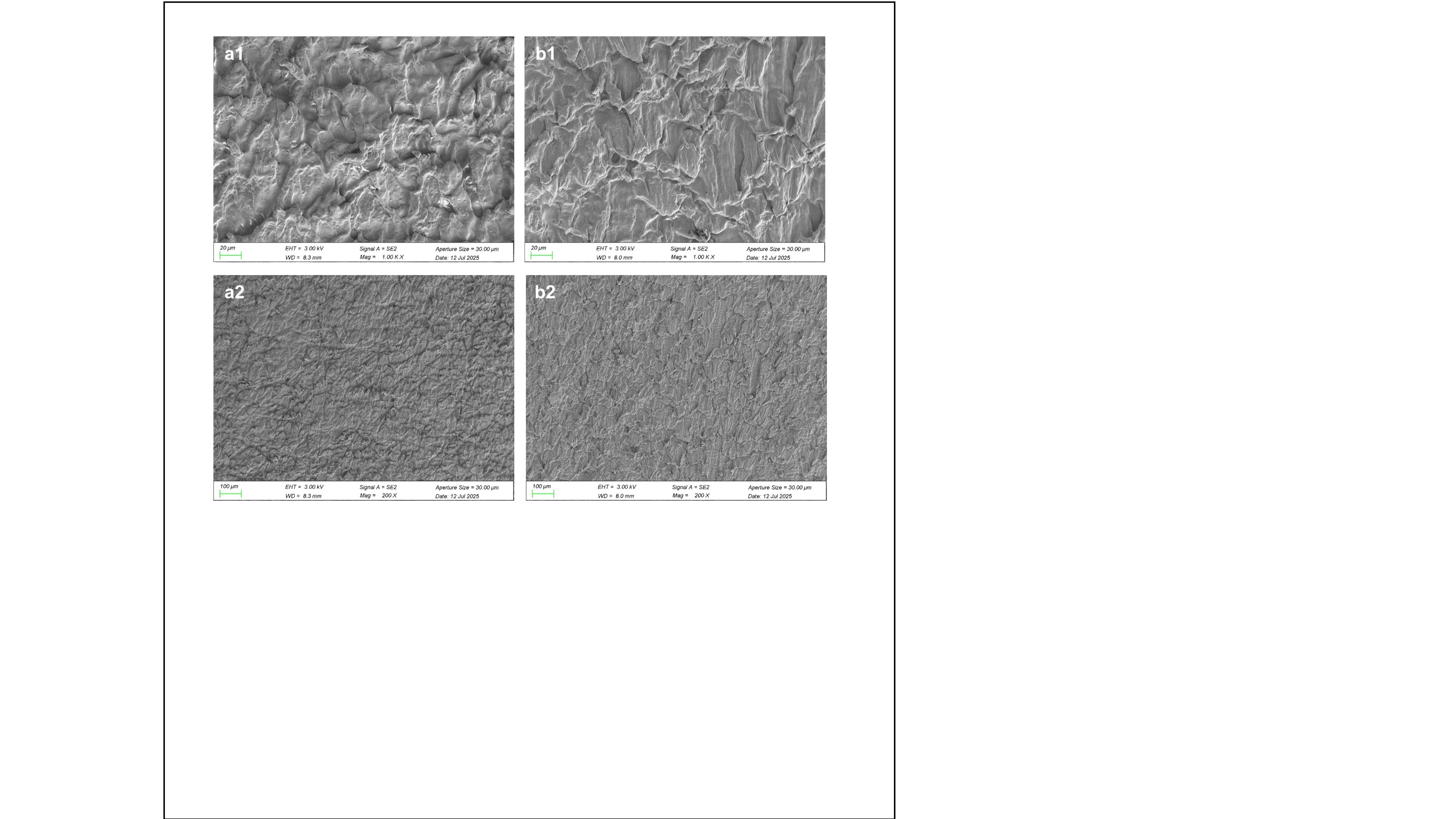


**Figure S5.** The SEM images of TPD gel samples peeled from electrode layers treated with 6-minute and 15-minute ultrasonic cavitation.


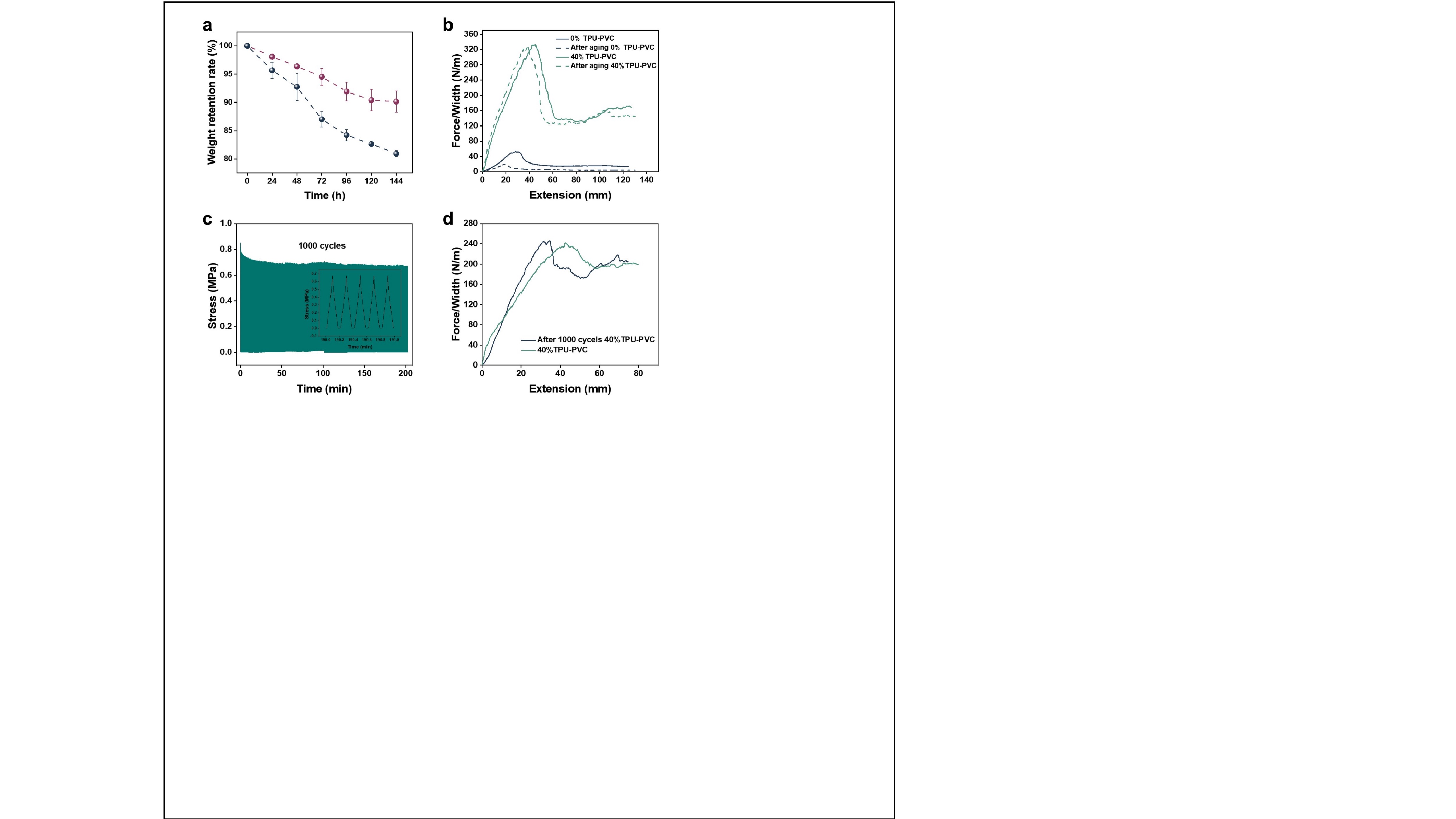


**Figure S6.** Accelerated aging tests and long-term cyclic stretching tests of C@T-TPD gel. (a) The variation of weight retention rate over time. (b) Force-extension curves of interfaces before and after aging. (c) The stress curve of 1000 cycles of tensile testing. (d) Force-extension curves of interfaces before and after 1000 cycles of tensile testing.


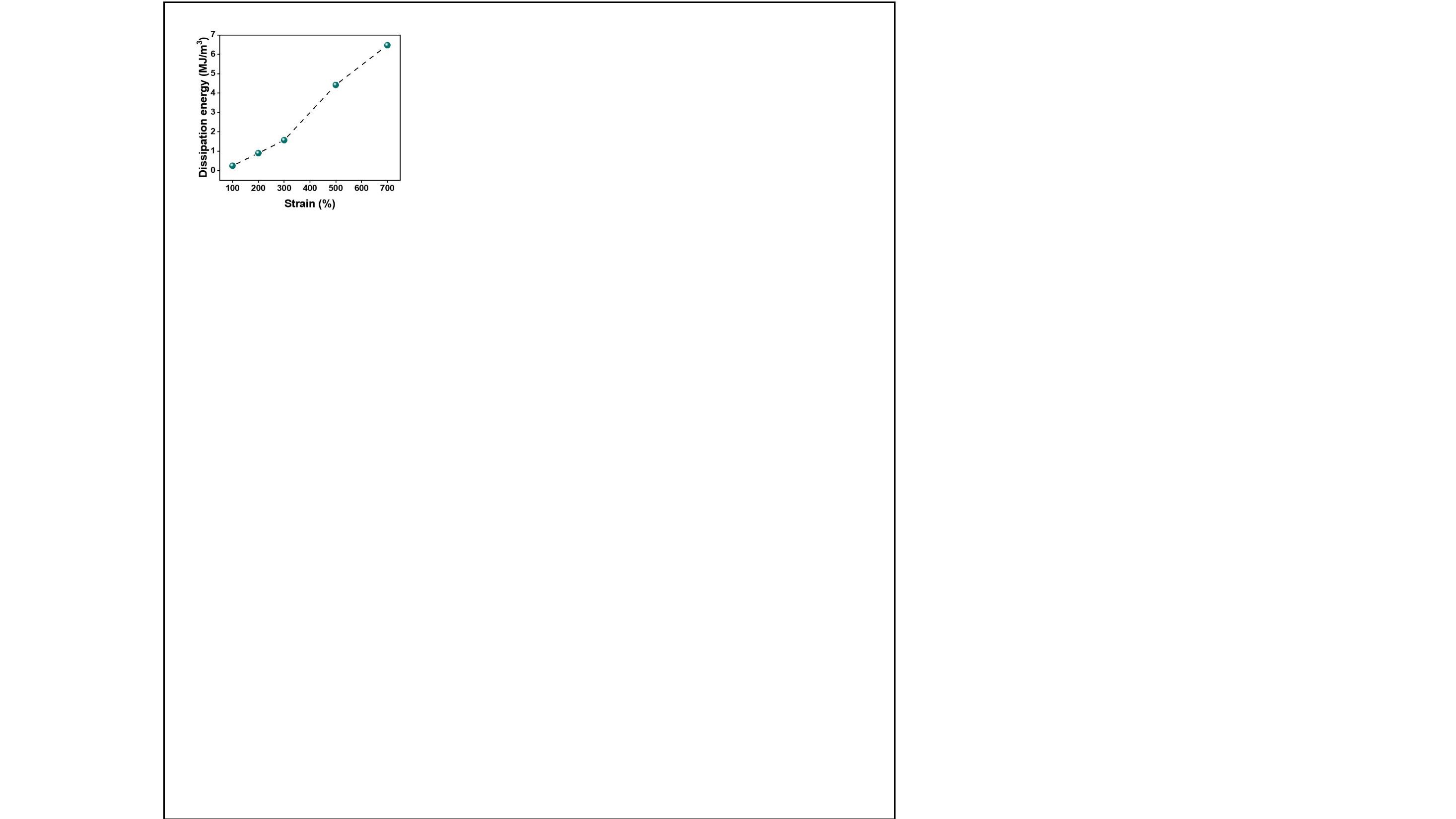


**Figure S7.** The dissipated energy during loading-unloading.


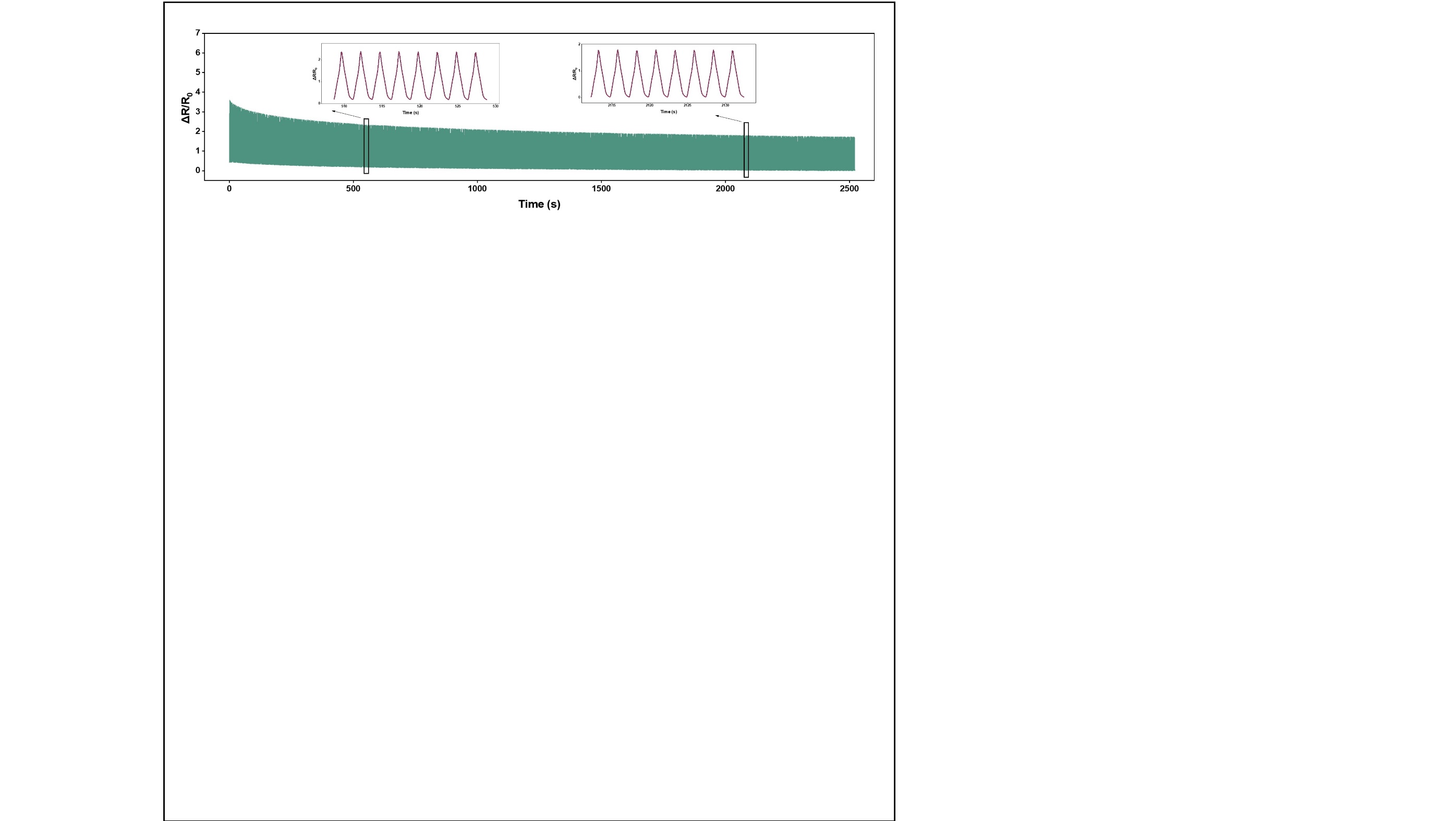


**Figure S8.** The sensor maintains stable performance with minimal fluctuation even after 1000 stretching cycles.


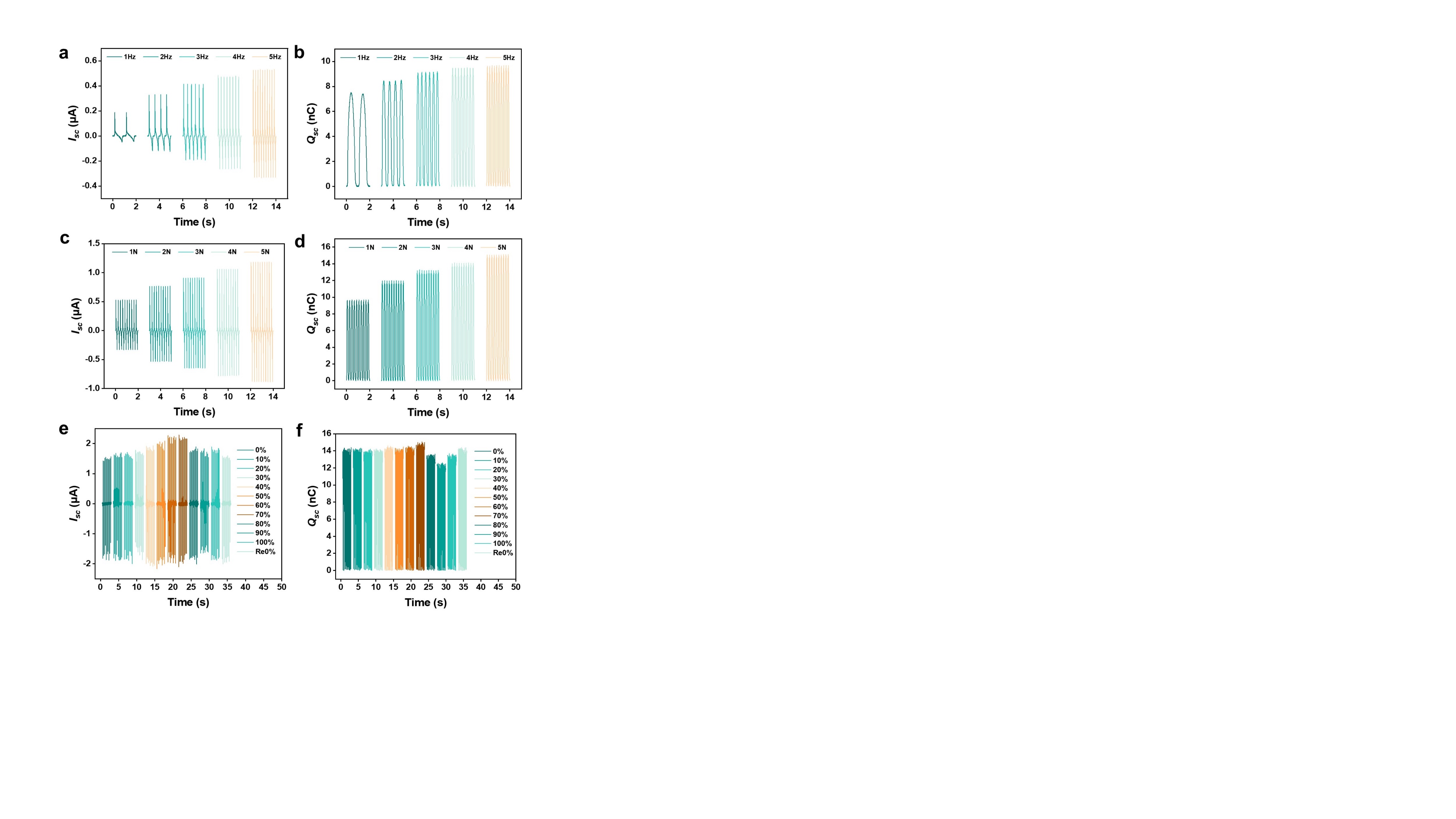


**Figure S9.** The corresponding short-circuit current and transferred charge under different test parameters.


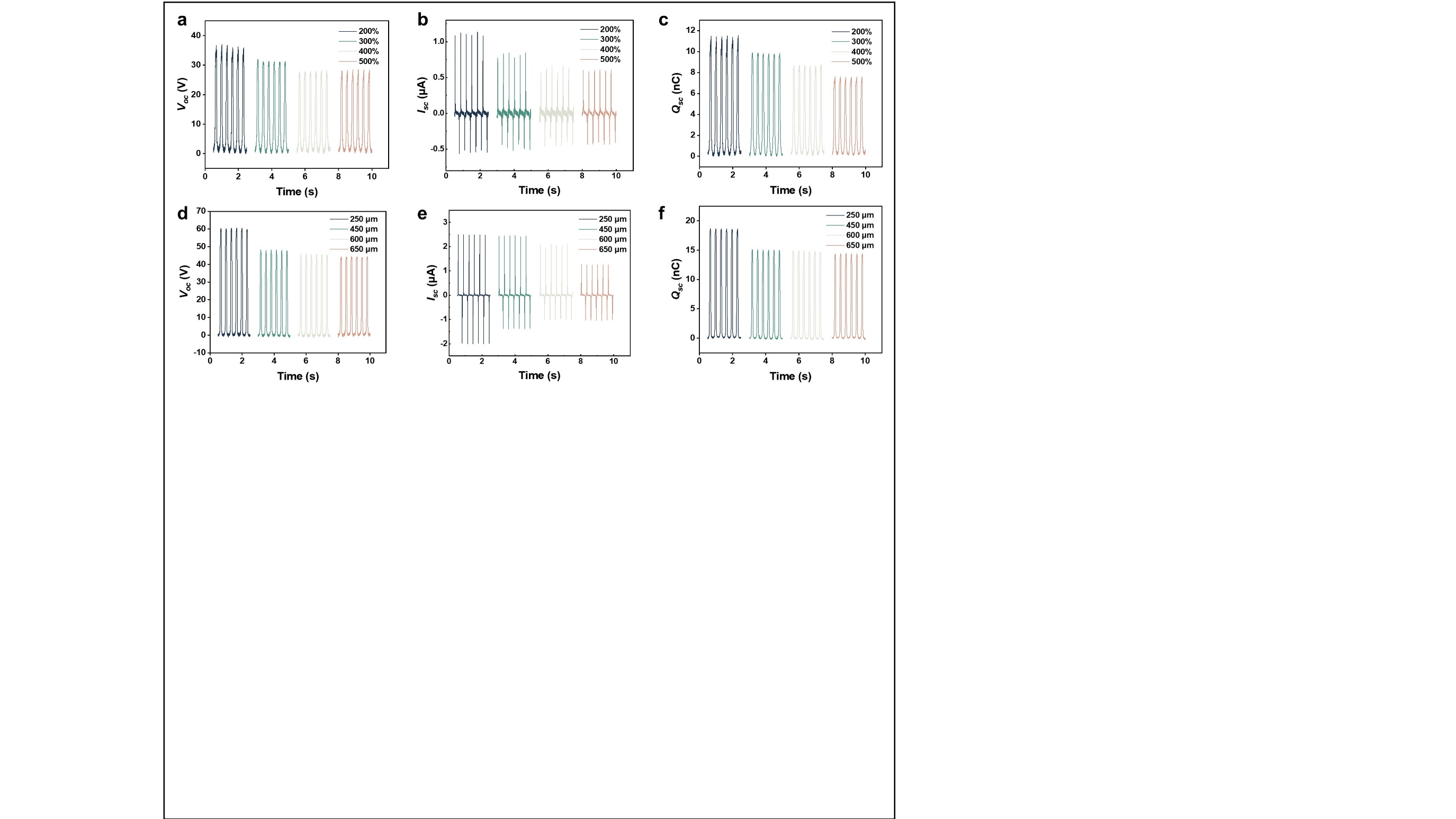


**Figure S10.** TENG output tests (a-c) across a wide strain range and (d-f) for different gel thicknesses.


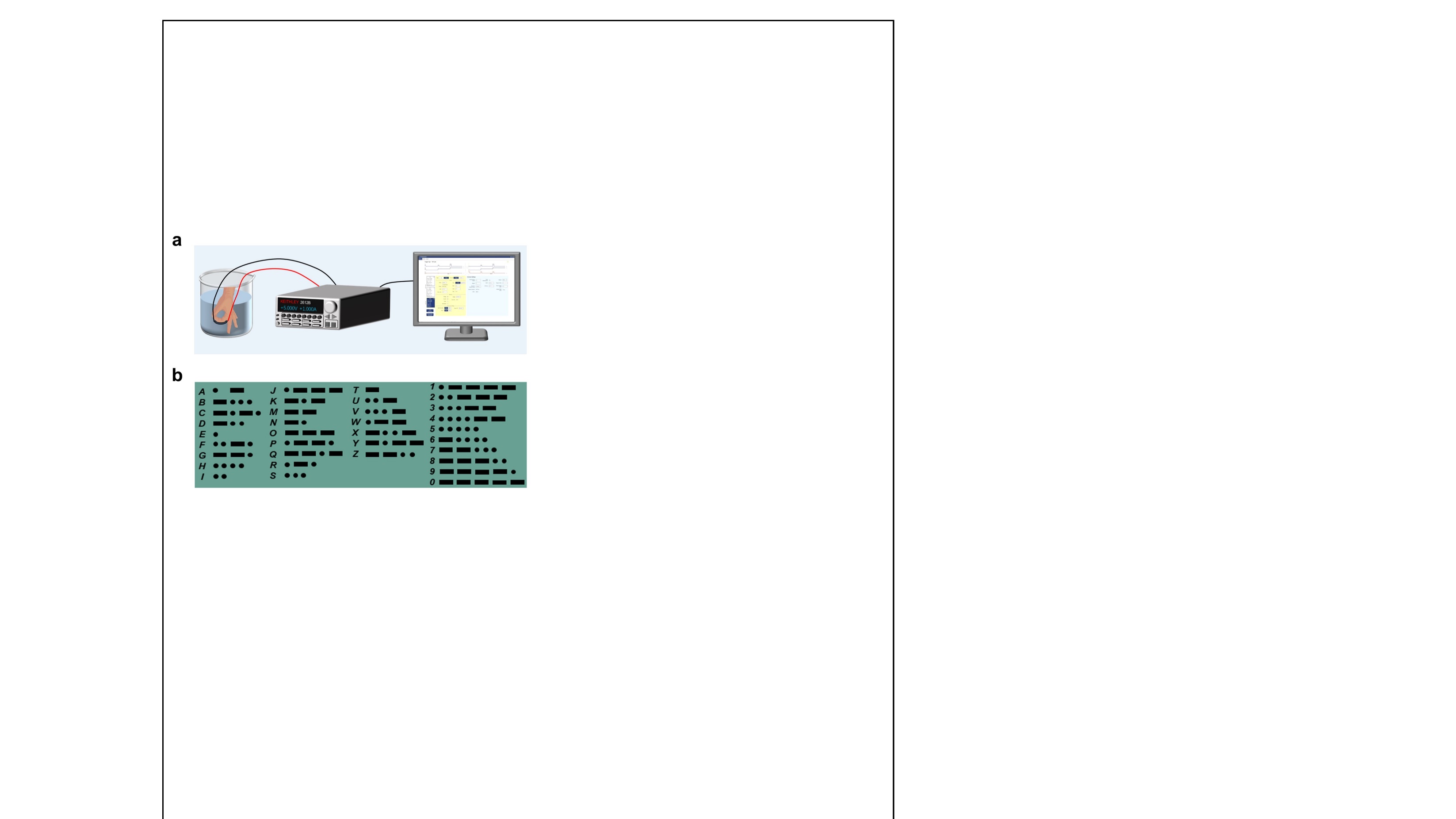


**Figure S11.** (a) Schematic diagram of underwater sensing test flow. (b) Electrical signal reflection of underwater finger bending.


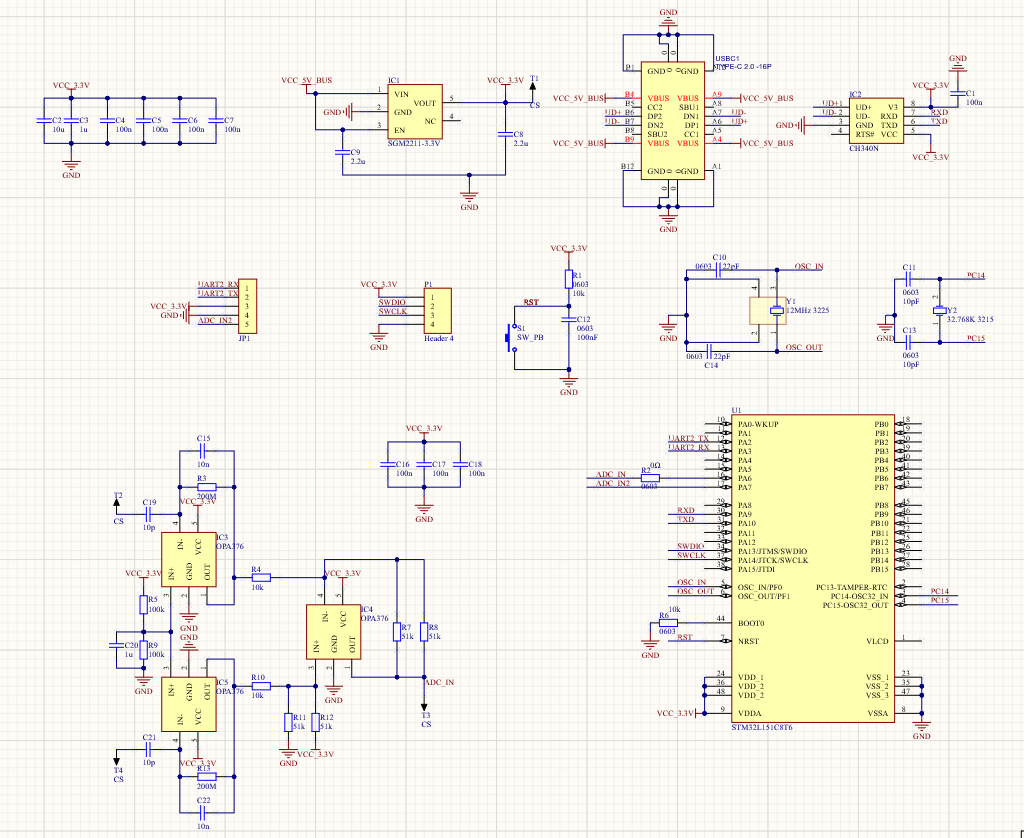


**Figure S12.** The voltage measurement circuit schematic of C@T-TPD gel-based TENG.

**Table S1** The performance comparison with other reported state-of-the-art methods.

| Materials | Strategy | Interfacial toughness (N/m) | Maximum strain (%) | Ref. |
| --- | --- | --- | --- | --- |
| Ionic polymer gel / Ecoflex | Gel banding | 46 | 375 | [21] |
| PVA hydrogel / PDMS | Physical interlocking & Gel banding | 60 (PDMS has fractured) | 250 | [22] |
| PAAM-alginate hydrogel / PDMS | Chemical bonding | 49 (PDMS has fractured) | 400 | [24] |
| PAM hydrogel / TPU | Chemical bonding | 33 | / | [25] |
| STAIC  hydrogel / PDMS | Chemical bonding | / | 350 | [26] |
| PAAM hydrogel / Ecoflex | Chemical bonding | 30 | 400 | [28] |
| **C@T / TPD gel** | **Multimodal banding** | **190** | **1110** | **This work** |
